# *In vitro* Colon Fermentation of Soluble Arabinoxylan Is Modified Through Milling and Extrusion

**DOI:** 10.3389/fnut.2021.707763

**Published:** 2021-08-25

**Authors:** Teresa Demuth, Veronica Edwards, Lea Bircher, Christophe Lacroix, Laura Nyström, Annelies Geirnaert

**Affiliations:** ^1^Laboratory of Food Biochemistry, Institute of Food, Nutrition and Health, ETH Zurich, Zurich, Switzerland; ^2^Laboratory of Food Biotechnology, Institute of Food, Nutrition and Health, ETH Zurich, Zurich, Switzerland

**Keywords:** arabinoxylan, prebiotic activities, food processing, gut microbiota, *in vitro* fermentation, SCFA

## Abstract

Dietary fibers such as arabinoxylan (AX) are promising food constituents to prevent particular diet-related chronic diseases because of their prebiotic properties. Arabinoxylan fermentation by the gut microbiota depends on the structural architecture of AX, which can be modified during food processing and consequently affect its prebiotic potential, but it is little investigated. Therefore, the aim of this study was to evaluate the effects of naturally occurring and processing-induced structural alterations of the soluble AX of wheat bran and rye flour on the *in vitro* human colon fermentation. It was found that fermentation behavior is strongly linked to the AX fine structure and their processing-induced modifications. The short-chain fatty acid (SCFA) metabolism, acidification kinetics, bacterial growth, and bacterial composition revealed that wheat bran AX (WBAX) was fermented faster than rye flour AX. Increased levels of bound phenolic acids resulting from processing were identified as the inhibiting factor for AX fermentation kinetics. Bacterial genera promoted by AX varied between AX source and processing type, but also between microbiota. Extruded WBAX promoted butyrate production and growth of butyrate-producing *Faecalibacterium* in the butyrogenic microbiota while it did not enhance fermentation and inhibited the growth of *Prevotella* in the propiogenic microbiota. We anticipate that the findings of this study are a starting point for further investigation on the impact of processing-induced changes on the prebiotic potential of dietary fibers prior to human studies.

## Introduction

The human gut microbiota represents a diverse ecosystem, which is populated by more than 10^14^ microorganisms harboring 100 times more genes than the human genome itself and, therefore, representing a tremendous metabolic potential (1). This dynamic population has a significant contribution to human health by acting against pathogens, shaping the intestinal epithelium, regulating the host immune system, and degrading undigested food such as dietary fibers (2). Besides other environmental factors, diet plays a key role in shaping and maintaining the microbiota composition and activity (3).

The intake of dietary fibers is elementary for a healthy diet and healthy gut microbiota. Plant-based polysaccharides are resistant to digestion in the human small intestine, but are fermented by the microbes present in the colon (4, 5). This prebiotic activity leads to the various health benefits of dietary fibers, such as increasing the formation of short-chain fatty acids (SCFA) and causing fecal bulking. However, not all dietary fibers are prebiotics as, according to the latest definition, they need to be selectively utilized by the host microorganisms conferring a health benefit (6, 7). In addition to fruits and vegetables, cereals such as rye, barley, oat, and wheat are an important source of dietary fibers in the Western human diet.

Among the common cereal grains, arabinoxylan (AX) is the primary non-starch polysaccharide. According to literature, both water-soluble and water-insoluble AX exhibit various beneficial functions in the body such as an increase in SCFA owing to its prebiotic effect (8). Although the water-soluble AX fraction only accounts for 1–2% of the total AX in grains, many of its reported health-promoting effects can be attributed to water-soluble AX. It is mainly localized in the cell walls of starchy endosperm, aleurone, and bran tissues (9). The contents and molecular fine structure, which can be evaluated by molecular weight (M_w_), sugar composition, and linkage pattern, depend on the AX source. Generally, AX consists of a linear (1–4)-β-D-linked xylopyranosyl backbone, substituted with α-L-arabinofuranosyl groups at positions O-3 and/or O-2 of the β-D-Xylp residues. In addition, phenolic acids, mainly ferulic acid, can be esterified on the C(O)-5 position of arabinose (10). Water-soluble wheat flour AX contains approximately one-third linked monosubstituted xylopyranosyl groups with two-thirds double substituted, whereas rye flour AX is significantly more singly substituted (11).

Apart from the naturally given structural alterations, food processing causes additional structural modifications of AX. The application of heat, pressure, or mechanical forces induces oxidative and hydrolytic reactions, which both modify the native chemical structure (12–15). Consequently, these structural modifications influence physicochemical properties and may further affect the prebiotic potential of AX (16, 17).

Only a fraction of the human gut bacteria is able to hydrolyse and ferment AX (18, 19). Previous work showed the proliferation of beneficial *Bifidobacterium, Lactobacillus, Prevotella* and *Bacteroides spp*. after AX fermentation. Induced growth can be explained by their ability to produce hydrolytic enzymes such as endo-1,4-β-D-xylanase, α-L-arabinofuranosidases, and β-D-xylosidases. This enzymatic digestion and the resulting metabolism of released monosaccharides lead to the production of beneficial SCFA metabolites and modulates the gut environment (20–22).Various studies have shown that the *in vitro* fermentation of AX is strongly linked to the molecular fine structure of AX (23, 24). Similarly, Tuncil et al. (17) hypothesized that subtle variations in dietary fiber structures may result in altered microbiota compositions. In particular, differences in M_w_ and the branching of soluble cereal AX affect SCFA production and bacterial growth during *in vitro* fermentation by human fecal microbiota (20, 25). A recent study by De Paepe et al. (26) confirmed that released linear oligosaccharides are fermented more rapidly than oligosaccharides with a more complex molecule structure, such as AX. From another perspective, De Paepe et al. (26) showed that the modification of wheat bran physicochemical properties allows control over the extent and rate of SCFA production. Another study presented that wheat bran fermentation was not influenced by a dry heat treatment (27). These studies focused on the correlation of process-induced changes with the physicochemical properties of wheat bran and its impact on *in vitro* fermentation, whereas the processing-induced changes of single prebiotic compounds, such as AX isolates, at a molecular level remain unknown (16, 28, 29). Thus far, the detailed relationship of processing-induced structural modifications of soluble AX and its effect on the prebiotic activity has not been fully elucidated yet.

Hence, the objective of this study was to investigate the effect of grain milling and extrusion on the *in vitro* colon fermentation of soluble AX isolated from wheat bran (WB) and rye flour (RF). For this purpose, the fine structure of milled and extruded WBAX and RFAX was characterized by the high-performance size exclusion chromatography-refractive index (HPSEC-RI), viscometer (VISC), right angle scattering (RALS), light angle scattering (LALS), high performance liquid chromatography-ultraviolet detection (HPLC-UV), and high-performance anion exchange chromatography-pulsed amperometric detection (HPAEC-PAD) and correlated with its prebiotic activity. The prebiotic activity of the processed AX was evaluated by means of an *in vitro* fermentation by two different cultivated human colon microbiota. Therefore, both the SCFA kinetics and microbiota composition were analyzed using quantitative PCR (qPCR) and 16S rRNA gene amplicon sequencing.

## Materials and Methods

### Materials

Amicase®, α-amylase (*Bacillus lichenformis*; Thermamyl 300 L), L-(+)-arabinose (≥99%), L-ascorbic acid (≥99%), caffeic acid (≥95%), cinnamic acid (≥99%), 3,5-dichloro-4-hydroxybenzoic acid (97%), para-coumaric acid (≥98%), ferulic acid (>99%), formic acid (HCOOH; ≥98%), D-(+)-glucose (≥99%), hydrogen peroxide (H_2_O_2_; 35%), para-hydroxybenzoic acid (>98%), iron(II) sulfate heptahydrate (FeSO_4_·7H_2_O; ≥99%), magnesium sulfate (MgSO_4_; ≥99%), meat extract, mucin from porcine stomach, pepsin from porcine gastric mucosa (250 U/ml), potassium chloride (KCl; ≥99%), protocatechuic acid (≥97%), sinapic acid (≥98%), sodium bicarbonat (NaHCO_3_; >99%), sodium hydroxide (NaOH, 50% in water), sodium nitrate (NaNO_3_; ≥99.5%), sodium phosphate dibasic dihydrate (Na_2_HPO_4_·2H_2_O; ≥98%), D-(-)-sorbitol (≥99%), syringic acid (≥95%), trifluoroacetic acid (CF_3_COOH; ≥99%), and vanillic acid (≥97%) were purchased from Sigma-Aldrich Chemie GmBH (Buchs, Switzerland). D-(+)-Galactose (≥99%), sodium azide (NaN_3_; >99%), and D-(+)-xylose (≥99%) were obtained from Fluka Chemie GmBH (Buchs, Switzerland). Hydrogen chloride (HCl; 37%) and sodium hydroxide (NaOH; ≥98%) were purchased from VWR International (Radnor, PA, USA). Amyloglucosidase (*Aspergillus niger*; 200 U/ml), wheat arabinoxylan (medium viscosity; ~95%), rye arabinoxylan (high viscosity; ~95%), lichenase (*Bacillus subtilis*; 1,000 U/ml), and protease (*Bacillus lichenformis*; 300 U/ml) were obtained from Megazyme (Bray, Ireland). Calcium chloride dihydrate, (CaCl_2·_2*H*_2_O; >99%), manganese chloride tetrahydrate (MnCl_2_·4H_2_O, >98%), sodium chloride (NaCl, >99%), and zinc sulfate heptahydrate (ZnSO_4_ 7 H_2_O, >99%) were obtained from Fischer Scientific UK Limited (Loughborough, UK). Bile salts were obtained from Oxoid AG (Pratteln, Switzerland). Bacto Tryptone™ was obtained from Becton, Dicksinson, and Company (Le pont de claix, France). Polyethlene oxide (PEO-24k; M_w_ = 24,063 g/moL, M_n_ = 23,618 g/moL, dn/dc = 0.132 ml/g, η = 0.4 dl/g) and dextran (T70K, M_w_ = 70,026 g/moL, M_n_ = 55,411 g/moL, dn/dn = 147 ml/g, η = 0.26 dl/g) were obtained from Malvern Instruments (Worcestershire, UK). The FastDNA™ SPIN KIT for soil was purchased from MPbio (Zurich, Switzerland). Water of MilliQ quality, H_2_O; ≥18.2 MΩ cm at 25°C, was used for aqueous solutions (Merck Millipore, Darmstadt, Germany).

### Methods

#### Arabinoxylan Processing and Extraction

SwissMill (a division of the Coop cooperative, Basel, Switzerland) kindly provided the native and extruded WB from *Triticum aestivum*. Milled WB sample material was additionally produced by ultra-centrifugal milling for the simulation of mechanical processing. For this purpose, a ZM200 ultra centrifugal mill (Retsch GmBH, Hann, Germany) was equipped with a 12-teeth rotor and a sieve with trapezoid holes of 0.5 mm. The Technical Research Centre of Finland (VTT Finland) kindly provided the native and extruded RF. Water-extractable arabinoxylan was extracted from the sources mentioned above using a previously published method (15). The freeze-dried material of multiple extractions was pooled and pulverized in a ball mill equipped with a 15-ml grinding bowl and two steel balls with a diameter of 15 mm for 1 min.

To compare the influence of impurities of AX extracts on the prebiotic potential, both wheat and rye fermentations were additionally performed with a commercial wheat and rye AX standard, namely, WAX and RAX, respectively. In both cases, Fenton oxidized WAX and RAX, namely, OXWAX and OXRAX, were additionally produced to simulate food processing-induced structural modification (30). For this purpose, WAX or RAX (132 mg) was dissolved in H_2_O (5 ml) for 1 h at 80°C and stirred at 20°C overnight. Following that, 50 μM FeSO_4_, 50 μM ascorbic acid, and 50 mM H_2_O_2_ were added in this order to give a final concentration of 2% AX (w/v). The Fenton oxidation was heated for 3 h at 80°C, then continued at room temperature with access to air for 24 h. The oxidation was done in triplicates and subsequently freeze-dried and pulverized analogously to the extracts. Structural features of both standards and the respective oxidized standard are shown in the Table 1.

**Table 1:** Structural features of differently processed wheat bran (WB) and rye flour (RF) arabinoxylan (AX) and wheat and rye AX standards (WAX, RAX) as well as the respective oxidized standard (OXWAX, OXRAX)^*^.

	**Native WBAX**	**Milled WBAX**	**Extruded WBAX**	**Native RFAX**	**Extruded RFAX**	**WAX**	**OXWAX**	**RAX**	**OXRAX**
M_w_ [kg/moL]	403 ± 41^a^	134 ± 11^b^	345 ± 21^a^	234 ± 18^i^	520 ± 50^ii^	330	6.28 ± 0.08	440	10.2 ± 0.1
Arabinoxylan [mg/g]	415 ± 10^a^	563 ± 30^b^	463 ± 42^c^	480 ± 11^i^	540 ± 21^ii^	950	950	900	900
Arabinose [mg/g]	135 ± 3^a^	173 ± 10^b^	141 ± 15^a^	134 ± 7^i^	161 ± 09^ii^	N/A	N/A	N/A	N/A
Xylose [mg/g]	280 ± 6^a^	391 ± 20^b^	322 ± 28^c^	330 ± 12^i^	370 ± 19^ii^	N/A	N/A	N/A	N/A
A/X ratio	0.483 ± 0.002^a^	0.441 ± 0.005^b^	0.436 ± 0.008^c^	0.41 ± 0.01^i^	0.44 ± 0.01^ii^	0.61	0.61	0.61	0.61
Glucose [mg/g]	9.4 ± 1.1^a^	5.7 ± 1.0^b^	28.9 ± 3.4^c^	2.10 ± 0.20^i^	5.2 ± 0.3^ii^	N/A	N/A	N/A	N/A
Galactose [mg/g]	10.3 ± 0.3^a^	6.4 ± 0.2^b^	4.4 ± 0.4^c^	–	–	N/A	N/A	N/A	N/A
Protein [mg/g]	50.7 ± 0.2^a^	49.3 ± 1.7^a^	48.9 ± 0.5^a^	24.0 ± 0.8^i^	14.0 ± 1.0^i^	N/A	N/A	N/A	N/A
Bound phenolic acids [mg/g]	2.4 ± 0.7^a^	4.0 ± 1.0^b^	4.0 ± 0.7^b^	6.0 ± 0.6^i^	6.67 ± 1.07^i^				

*An in-depth characterization of the WBAX was published earlier (15)*.

* *Note that letters indicate significant differences corresponding to α = 0.05 among the differently processed samples, compared means were native, milled, and extruded WBAX as well as native and extruded RFAX*.

#### Extract and Monomeric Sugar Composition

The monomeric sugar composition of the extracts was determined according to Demuth et al. (15) by high-performance anion-exchange chromatography with pulsed amperometric detection (HPAEC-PAD) after hydrolysis with trifluoracetic acid. The 10-mg sample materials were hydrolyzed with 2.5 ml 2 M TFA for 4 h at 100°C. The HPAEC-PAD separation was based on a procedure by Hardy et al. (31) and Rohrer et al. (32) with modifications. A Dionex™ ICS-5000+ Capillary HPIC™ System with a Dionex™ CarboPac™ PA1 IC column (Thermo Fischer Scientific AG, Basel, Switzerland) maintained at 26°C was used. The injection volume was 10 μl and the flowrate was 1 ml/min. Eluent (A) consisted of water and eluent (B) had 200 mM of NaOH. The gradient started with 92% (A) for 20 min. After 20 min, the gradient was changed to 100% (B) for 10 min, and finally re-equilibrated for 8 min with 92% (A). For the pulsed amperometric detection, replaceable gold electrodes were used in waveform A adapted from the Dionex technical note 21. All samples were measured in quintuplicates. L-(+)-arabinose and D-(+)-xylose as well as D-(+)-glucose and D-(+)-galactose were externally calibrated using a 13-point calibration from 1.25 to 100 μg/ml with 10 μg/ml of D-(–)-sorbitol as internal standard. Data processing was done in Chromeleon 7 (Thermo Fischer Scientific AG, Basel, Switzerland).

#### Nitrogen Content

Elemental nitrogen (% N) content in the extracts was quantified in triplicates using a Flash EA 1112 Series elemental analyzer (Thermo Italy, former CE Instruments, Rhodano, Italy) coupled to a Finnigan MAT Delta^plus^XP isotope ratio mass spectrometer (Finnigan MAT, Bremen, Germany) as described by Werner et al. (33) and Brooks et al. (34). Subsequently, the protein content was calculated using a conversion factor of 5.7 for grains (35).

#### Phenolic Acid Determination

The bound phenolic acids were determined in quadruplicates by reversed-phase high performance liquid chromatography (RP-HPLC) with an ultraviolet detector (UV) as published earlier (15) using 10 mg of AX extracts. The eluted phenolic acids were detected at 254, 280, and 325 nm and processed by Chromeleon 7 (Thermo Fischer Scientific AG, Basel, Switzerland).

#### M_w_ Analysis

The weight average molecular weight (M_w_) of the AX extracts was determined using high performance size exclusion chromatography (HPSEC) coupled with triple detection (15). The system consisted of OMNISEC resolve (OMNISEC, Malvern Panalytical Ltd., Malvern, United Kingdom) coupled to the multi-detector module OMNISEC reveal (OMNISEC, Malvern Panalytical Ltd., Malvern, United Kingdom) including an RI, RALS (90°), LALS (7°), and a VISC detector. Two A6000M columns with an exclusion limit of 20,000,000 g/moL (Malvern Panalytical Ltd., Malvern, UK) were maintained at 35°C with a flowrate of 0.7 ml/min using an aqueous mobile phase with 0.1 M NaNO_3_/0.02% NaN_3_. The injection volume was 100 μl, and the elution was recorded for 42 ml. Polyethylene oxide calibration standard (2.498 mg/ml, dn/dc 0.132 ml/g, M_w_ 23.85 kg/moL) and dextran verification standard (2.433 mg/ml, dn/dc 0.148 mL/g, M_w_ 70.03 kg/moL) solutions were prepared in an aqueous solvent with 0.1 M NaNO_3_/0.02% NaN_3_. Arabinoxylan isolates were dissolved equally by heating them to 80°C under constant stirring for 30 min, with a final concentration of 1 mg/ml. Subsequently, the samples were stirred overnight at 20°C and filtered using 0.45-μm nylon filters. The system was calibrated with a one-point calibration approach using a narrow PEO standard with known parameters. The samples were measured in triplicates and the data evaluation was performed with the Malvern software OMNISEC V10.30. The refractive index increment (dn/dc) of 0.132 ml/g was determined by HPSEC using WAX and RAX as AX reference standards.

#### *In vitro* Colon Fermentation of AX

The fermentation of various soluble WBAX and RFAX by colon microbiota was determined by an *in vitro* colon high-throughput batch fermentation using two different *in vitro* cultivated proximal colon microbiota (CM 1 and CM 2). The focus of this study was on the impact of processing-induced structural changes of AX on its colonic fermentation; therefore, the hydrochloric acid predigestion of extracts prior to the fermentation was not performed as it may have additionally modified the structure of AX, preventing the direct processing-structure-function correlation (36). The microbiota were derived from two independent stable PolyFermS systems, a continuous colon fermentation model, which were inoculated with the immobilized fecal microbiota of healthy human adults and are designed to continuously cultivate the proximal colon microbiota akin to donor profile (37, 38). The fecal samples for initiating the PolyFermS system were donated by two healthy individuals (females, aged 27 and 28) with Western-style diets who did not receive any antibiotics or probiotics for at least 3 months before donation. The Ethics Committee of ETH Zürich exempted this study from review because the sample collection procedure was not performed under conditions of intervention. Informed written consent was obtained from the fecal donors. The donor fecal and *in vitro* cultivated proximal colon microbiota profile is provided in the (Supplementary Table 1).

*In vitro* colon microbiota fermentations were performed in a Macfarlane-based medium for the cultivation of the human colon microbiota (39). A double-concentrated Macfarlane medium without fibers containing 3 g/L amicase, 5 g/L Bacto™ tryptone, 1.5 g/L meat extract, 4.5 g/L yeast extract, 4 g/L mucine from porcine stomach, 0.4 g/L bile salt, 3 g/L potassium dihydrogen phosphate, 9 g/L sodium bicarbonate, 4.5 g/L sodium chloride, 4.5 g/L potassium chloride, 0.65 g/L magnesium sulfate, 100 mg/L calcium chloride, 200 mg/L manganese chloride, 5 mg/L iron sulfate, 100 mg/L zinc sulfate, 5 mg/L, and 5.7 ml/L (v/v) of a volatile fatty acid mix (acetate, propionate, isovalerate, isobutyrate, valerate) with a pH of 6.8 was prepared (40).

The media were filled anaerobically into serum flasks, sealed, and then autoclaved. A sterile-filtered vitamin solution was added to the medium prior to the experiment (41). Predigesting of the evaluated AX extracts was generally prevented to exclude any additional structural modifications to the one induced by processing. The AX solutions were heated to 80°C for 30 min, stirred overnight, and filter sterilized using 0.22 μm PTFE filters (BGB Analytik AG, Boeckten, Switzerland). Subsequently, AX solutions were added to the media to obtain a final concentration of 5 mg/ml AX and a normal concentrated Macfarlane medium (39). The determined AX purities of the different AX extracts were considered in the preparation of AX solutions targeting a final concentration of 5 mg/ml in all AX solutions (see Table 1 AX content). The investigated dose (5 mg of soluble AX/ml) mimics a dietary intake of 1 g of soluble AX/day when taking into account the average volume of the proximal colon (200 ml (42)) and assuming no upper digestive tract AX degradation and uptake. This dose is within the range of a Western human adult diet based on an average whole grain intake of 50 g/day (43) and a soluble AX content of approximately 2.2% (10) or up to 1–2% (10, 15) in rye flour or wheat bran, respectively.

The mixture of fiber and medium solution was added to 24-well plates and inoculated with an *in vitro* cultivated proximal colon microbiota at an inoculation rate of 1% (v/v) and final fermentation volume of 2 ml. A negative control was included consisting of a fiber-free Macfarlane medium. All manipulations were done in an anaerobic tent (10% CO_2_, 5% H_2_, 85% N_2_) (Coy Laboratories, MA, USA) and the well-plates were incubated anaerobically for 0, 6, 12, 24, or 48 h at 37°C in the dark. Each treatment per *in vitro* colon microbiota and per time point was performed in technical quadruplicates. At each time point, fermentation of one 24-well plate was stopped and sampled for pH, SCFA, and bacterial quantification.

The fermentation kinetics was assessed by pH measurement and SCFA quantification over time and correlated with changes investigated in bacterial growth and composition at 12 h and 24 h.

#### SCFA Quantification

The fermentation metabolites acetate, propionate, butyrate, isobutyrate, lactate, succinate, valerate, iso-valerate, and formate were quantified. Therefore, an aliquot of 1 ml at each sampling point was centrifuged (13,000 cfm for 10 min at 4°C) and the supernatant was then filtered using an 0.45 μm PTFE (Phenomenex Helvetia GmbH, Basel, Switzerland) filter. The samples were then analyzed using an HPLC (Merck-Hitachi, Selm, Germany) equipped with a Cation-H refill cartage (Bio-Rad Laboratories AG, Reinach, Switzerland) (30 × 4.6 mm) connected to an Aminex® (Bio-Rad Laboratories AG, Reinach, Switzerland) HPX-87H (300 × 7.8 mm) column and a refractive index detector (Thermo Fisher Scientific AG, Pratteln, Switzerland). The injection volume was 40 μl. The mobile phase used was 10 mM H_2_SO_4_ with a flow rate of 0.6 ml/min at 40°C under isocratic conditions. The metabolites were quantified by external calibration.

#### Microbial Community Analysis

The microbial community was analyzed by qPCR and Illumina next generation 16S rRNA gene amplicon sequencing. The analysis was carried out on randomly selected technical triplicates or all quadruplicates from timepoints 12 and 24 h. Genomic DNA was extracted from the pellet of 1 ml of the fermentation sample and performed according to the procedure of the FastDNA™ SPIN KIT for soil purchased from MPbio (Zurich, Switzerland). Total DNA concentration (ng/μl) and purity were determined by spectrophotometry using the Nanodrop (Nanodrop ND 1000, Thermo Scientific, Wilmington, DE, USA). Then, the DNA extracts were diluted to a DNA concentration of 20 ng/μl in nuclease-free molecular grade water and stored at 4°C until any further downstream analysis.

Quantitative real-time PCR of the 16S rRNA gene for total bacteria [primer Eub338F and Eub518R), *Lactobacillus/Leuconostoc/Pediococcus* (primer F_Lacto 05, F_Lacto 04)], and *Bifidobacteria* (primer Bif F and Bif R) were performed in triplicates (44–46). The qPCR assays were performed in triplicates in 10 μl using 2 μl of diluted genomic DNA, 5 μl of 2X SensiFast SYBR No-ROX Mix (Bioline, Luckenwalde, Germany), and 500 nM of each forward and reverse primer. The analysis was performed in a Roche LightCycler 480 II (Roche Diagnostics, Rotkreuz, Switzerland). Reactions were pre-incubated at 95°C for 3 min, followed by 45 cycles at 95°C for 5 s and 60°C for 30 s, and then a melting curve analysis. For quantification, a dilution series of standards was obtained by amplification of the linearized plasmids containing the gene of a representative bacterial species belonging to the target group and included in each run. Primer specificity and verification of the presence of the desired amplicon were determined by melting curve analysis. The PCR efficiency (%) was calculated from the slope of the standard curve of each qPCR assay. Assays with an efficiency of 80–110% (slope of 3.2–3.9) were included in the data analysis. The gene copy number of the qPCR results were transferred to numbers of bacteria/ml by correcting for the median 16S rRNA gene copy number based on the Ribosomal RNA Database (47).

The microbiota community profiling was performed using 16S rRNA gene amplicon sequencing by StarSEQ (Mainz, Germany). The bacterial compositions of the differently treated WBAX and RFAX samples from timepoints 12 and 24 h were determined using tag-encoded 16S rRNA gene MiSeq-based (Illumina, CA, USA) high-throughput sequencing. The V4 region of the 16S rRNA gene was amplified with modified primers, namely, 515 F (TATGGTAATTGTGTGNCAGCMGCCGCGGTAA) and 806 R (AGTCAGTCAGCCGGACTACHVGGGTWTCTAAT). One MiSeq cell and the V2 2 × 250 bp paired end Next Tera chemistry supplemented with 20% of PhiX was utilized.

The raw sequence data have been submitted to European Nucleotide Archive (ENA) database with accession number PRJEB44740. Raw data were processed using the DADA2 R package [version 1.14.1, (48)] to obtain exact amplicon sequence variants (ASVs). Forward and reverse reads were truncated after 170 and 160 nucleotides, respectively. After truncation, reads with expected error rates higher than three and four for forward and reverse reads, respectively, were removed. After filtering, error rate learning, ASV inference, and denoising, reads were merged with a minimum overlap of 40 bp. Chimeric sequences were identified and removed. Taxonomy was assigned to ASVs using DADA2 against the SILVA database (v138) (49). The open-source bioinformatics pipeline Quantitative Insight Into Microbial Ecology (QIIME2) was used for subsequent analysis, including alpha and beta diversity (50).

#### Statistical Analysis

The ANOVA one-way statistical analysis with a significance level of 0.05 was performed to identify significant differences between means in structural analysis and SCFA concentration considering the assumptions required for parametric testing. *Post-hoc* analysis was carried out using Tukey HSD in Origin pro 2019 (OriginLab Corporation, MA, USA). Comparisons were performed among differently processed extracts and between oxidized and non-oxidized standards. Pairwise Kruskal–Wallis analysis was performed on alpha diversity metrics to identify significant differences between treatments in QIIME2. Permutational multivariate ANOVA (PERMANOVA) was performed based on weighted and unweighted UniFrac distance matrices followed by pairwise tests to identify the significant differences between treatments in QIIME2. To identify the significant differences in genus composition between treatments after 12 or 24 h fermentation, the DESeq2 method was applied with the DESeq and phyloseq package (51, 52) in R, version 4.0.4 (53).

## Results

This study was designed to investigate the effect of grain milling and extrusion on the *in vitro* colon microbiota fermentation of soluble AX isolated from WB and RF. Soluble AX was extracted from native, milled, and extruded WB, and from native and extruded RF. Furthermore, commercially available soluble AX standards originating from RAX and WAX were incorporated in all experiments to exclude the side effects of impurities in the generated extracts. In addition, these standards were Fenton oxidized to chemically simulate processing-induced modifications (OWAX and OXRAX). Finally, the naturally occurring and processing-induced structural changes of soluble AX were correlated with the fermentation behavior of two cultivated human colon microbiota (CM) to define a structure–function relation. For this purpose, the obtained AX extracts were characterized by the structural features M_w_, sugar composition, arabinose to xylose (A/X) ratio, and bound phenolic acids content.

### Structural Characterization of Differently Processed Soluble Arabinoxylan

In this study, M_w_ was determined by HPLC-RI/RALS/LALS/VISC. The M_w_ of native RFAX (234 ± 18 kg/moL) increased significantly by 100% through extrusion (520 ± 50 kg/moL) suggesting solubilization of insoluble AX (Table 1). In contrast, the extrusion of WB did not cause a significant change in M_w_, while the milling of WB induced a significant M_w_ reduction from 403 ± 41 kg/moL (native WBAX) to 134 ± 11 kg/moL (milled WBAX). The Fenton oxidation caused a significant M_w_ degradation through chain scissions in both WAX and RAX (Table 1), while the oxidation of RAX reduced the M_w_ from 440 kg/moL to 10.2 ± 0.1 kg/moL and an even stronger degradation was observed for WAX from 330 kg/moL to 6.28 ± 0.8 kg/moL.

The monomeric sugar composition of the WBAX isolates was determined after complete hydrolysis by HPAEC-IPAD. The varying extract purity for AX was considered in the subsequent fermentation experiment. The arabinose substitution changed significantly through processing (Table 1). The solubilisation of slightly branched high M_w_ AX during extrusion significantly increased the A/X ratio of rye flour from 0.41 ± 0.01 to 0.44 ± 0.01. In contrast, the A/X ratio of native wheat bran AX decreased significantly through both milling and extrusion. Moreover, the degree of substitution varied significantly between WBAX and RFAX. Generally, native RFAX (0.405 ± 0.009) presented a lower A/X ratio than native WBAX (0.483 ± 0.002), which indicated a less substituted structure. The soluble AX extracted from all three WB samples contained both glucose and galactose, whereas RFAX samples only contained glucose. In both cases, extrusion increased glucose content significantly. However, the glucose concentration in RFAX was lower than in the WBAX samples, even after extrusion with maximal 5.2 ± 0.3 mg/g. The highest glucose content was found for extruded WBAX with 28.9 ± 3.4 mg/g, which is in line with a study of Ralet et al. (54), who found that extrusion enhances the glucoarabinoxylan solubilization from the pericarp layer of wheat. Wheat bran AX samples had equal galactose concentrations. The presence of galactose can be explained by the co-extraction of residual water-soluble arabinogalactanpeptides (55). We observed the release of galactose through bacterial enzymatic degradation during the *in vitro* colon fermentation of WBAX, confirming the above structural data (data not shown).

The protein content among the extracts evaluated ranged between 1 and 5% in WBAX and RFAX. As a specific structural feature of AX, the total quantity of bound phenolic acid was determined in the extracts obtained. In general, RFAX contained significantly more bound phenolic acids than WBAX. For both sample types, processing increased the content of bound phenolic acids, which may be explained by the solubilization effect of high M_w_ AX with a rather complex molecular structure. However, it seems that extrusion partially degraded the liberated phenolics. Both ferulic and sinapic acid were the only two phenolic acids identified in the samples, whereas ferulic acid was dominated (data not shown).

### *In vitro* Colon Microbiota Fermentation of Differently Processed AX

The *in vitro* colon microbial fermentation of the different soluble AX extracts was evaluated over 48 h by means of pH change, SCFA production, and microbial community profiling. The two cultivated microbiota (CM) were representative for a healthy human proximal colon microbiota and differed in abundant bacterial families (Supplementary Table 1). Cultivated microbiota 1 was characterized by high levels of *Prevotellaceae* (56%), *Lachnospiraceae* (17%), and *Ruminococcaceae* (9%) while CM 2 was characterized by high levels of *Bacteroidaceae* (39%), *Lachnospiraceae* (22%), and *Ruminococcaceae* (14%). The two complementary CMs fermented the differently processed AX samples at different rates and to different extents.

#### AX Source and Processing Impacted pH Acidification Kinetics

**Figure 1:**
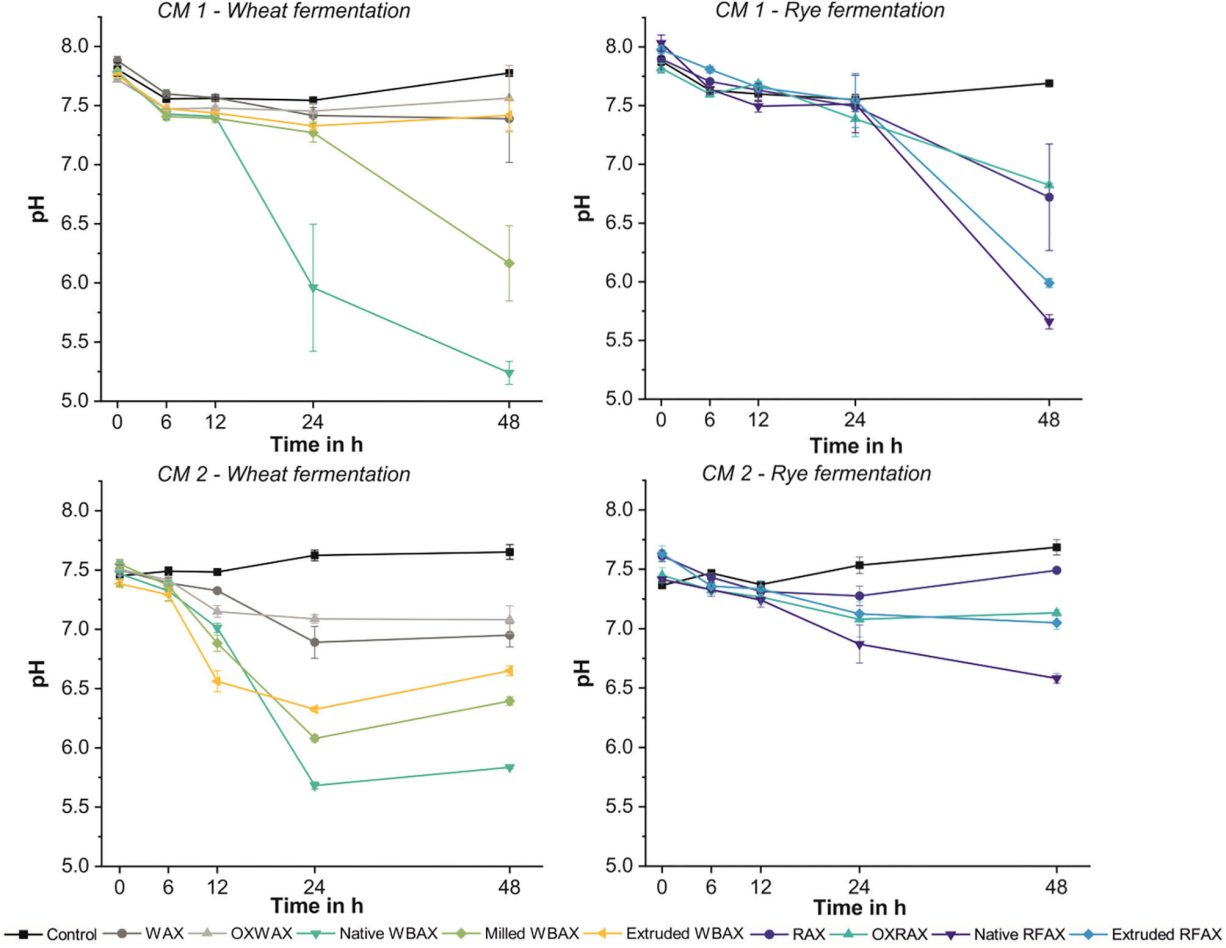
pH acidification during 48 h *in vitro* fermentation of differently processed WBAX and RFAX by colon microbiota (CM) 1 and CM 2 expressed as average ± stdev pH (*n* ≥ 4).

The pH kinetics revealed remarkable differences between the two AX sources, namely, RFAX and WBAX, and, more importantly, between the differently processed AX extracts (Figure 1). Generally, CM 1 presented a stronger acidification for both WBAX and RFAX than CM 2. In both fermentations, differently processed WBAX resulted in a stronger acidification than the RFAX. Commercial and oxidized AX standards incorporated in the experiment caused only a slight pH decrease compared to the extracts. Among the different treatments, both native RFAX and native WBAX showed the strongest acidification over 48-h fermentation with an average delta pH of −2.4 (CM 1) and −0.8 (CM 2) for RFAX and −2.5 (CM 1) and −1.6 (CM 2) for WBAX. The fermentation of milled WBAX resulted in a stronger pH decrease than extruded WBAX in both CMs. Interestingly, the extruded WBAX did not induce an acidification in CM 1 (delta pH of −0.2), while it did in CM 2 (delta pH of −0.7). Notably, the WBAX fermentation by CM 2 microbiota showed an increase in pH during the final fermentation period (24–48 h), which is an indication for completed carbohydrate fermentation followed by proteolytic fermentation and concomitant ammonia formation (56).

#### SCFA Production Varied Between Microbiota, AX Sources, and Processing Methods

**Figure 2:**
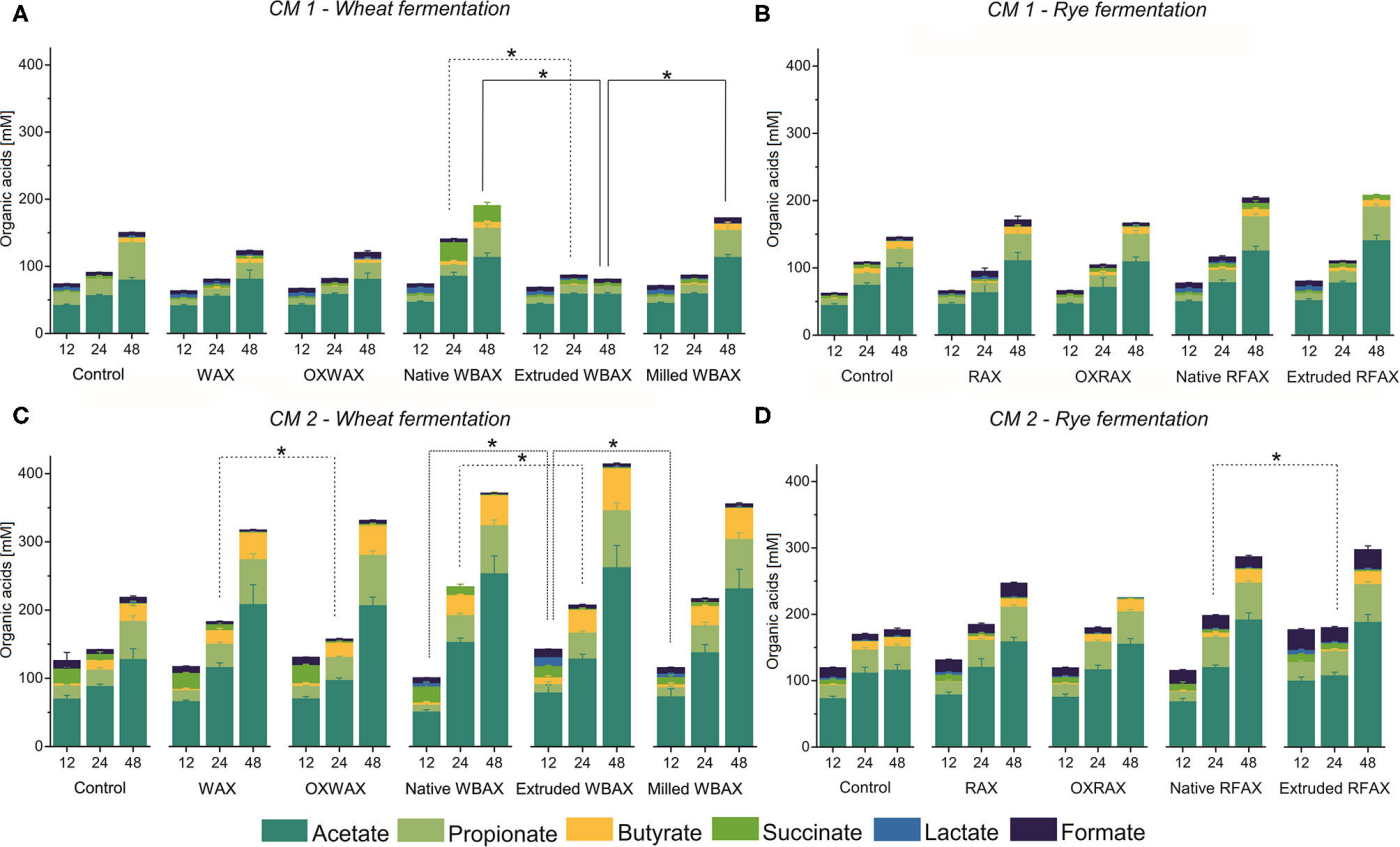
Microbial short-chain fatty acid (SCFA), lactate, and succinate production kinetics during 48 h *in vitro* fermentation of differently processed soluble WBAX and RFAX by CM 1 **(A,B)** and CM 2 **(C,D)**. Data are expressed as average ± st dev (*n* ≥ 4). Branched SCFA deriving from protein fermentation are displayed in the (Supplementary Figure 3). Significant differences in total organic acids concentration between differently processed AX are indicated at 12, 24, and 48 h, and correspond to α = 0.05(*).

**Figure 3:**
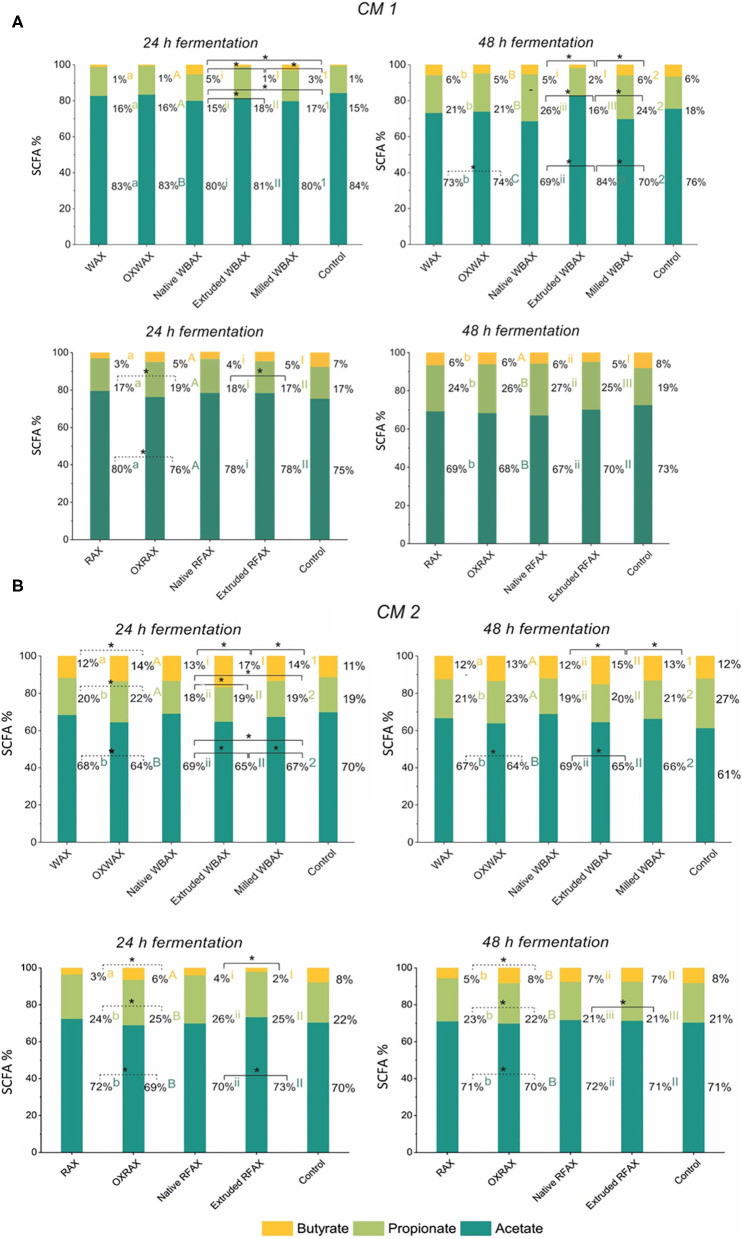
Relative abundance of the end metabolites acetate, butyrate, and propionate after 24 and 48 h *in vitro* fermentation of differently processed WBAX and RFAX by CM 1 **(A)** and CM 2 **(B)**. Different letters indicate the significant differences between average profile at 24 and 48 h for the different AX treatments. Lines indicate significant differences between the differently processed AX samples at 24 or 48 h. All significant differences correspond to α = 0.05(*).

Overall, the metabolite levels increased during the 48 h fermentation in both CMs regardless of AX source and treatment except for extruded WBAX in CM 1 (Figure 2). The SCFA acetate, propionate, and butyrate were the most abundant metabolites produced. Next to acetate, the main SCFA in CM 1 was propionate and, in CM 2, butyrate (Figure 3). The CM 2 microbiota generally produced more SCFA, lactate, and succinate than CM 1 microbiota with total levels ranging from 223.1 ± 10.4 (control) to 410 ± 50 mM (extruded WBAX) (Figures 2A,B) compared to 111 ± 12 and 196 ± 15 mM formed by CM 1 (Figures 2C,D). Moreover, the kinetics of the metabolite production and SCFA profile varied among the CMs evaluated. The majority of SCFA formed during the fermentation of WBAX and RFAX by CM 1 occurred after 24 h of incubation. In contrast, CM 2 produced significant SCFA levels already after 12 h of incubation. The enhanced fermentation rate by CM 2 is reflected in a faster acidification and a significantly higher SCFA formation combined with BCFA production observed for WBAX (Figure 1 and Supplementary Figure 3).

For the propiogenic CM 1 microbiota, differences in the production kinetics of intermediates (formate, lactate, and succinate) and end metabolites (acetate, propionate, and butyrate) between WBAX and RFAX and among differently processed AX were observed (Figure 2 and Supplementary Figure 2). Generally, native WBAX induced a significantly higher production of end metabolites compared to extruded and milled WBAX. In particular, extruded WBAX showed little increase in end metabolites over time. Furthermore, the distinct production and conversion patterns of the intermediate metabolites formate, lactate, and succinate could be observed during the 48 h fermentation of the different WBAX samples. Native WBAX induced a high production of succinate (28 ± 5 mM) at 24 h compared to extruded and milled WBAX (6.8 ± 0.6 mM and 6 ± 0.7 mM, respectively). Both native and milled WBAX resulted in a slightly higher lactate production at 12 h (8.7 ± 0.3 mM and 6.5 ± 0.2 mM, respectively) compared to extruded WBAX (5.3 ± 0.2 mM). In contrast to the WBAX fermentation, the total end metabolite production of the native and extruded RFAX fermentations were comparable (Figure 2B). However, distinct differences in metabolite composition were detectable over the progressing fermentation. During the fermentation of extruded RFAX, formate was faster metabolized or less accumulated than in native RFAX. Noteworthy, butyrate production was slower with RFAX samples compared to the negative control medium fermentation. Furthermore, differently processed WBAX and RFAX induced differences on the SCFA profile at 24 h, and WBAX also at 48 h (Figure 3A). Compared to native and milled WBAX, extruded WBAX resulted in relatively less propionate and butyrate at 48 h.

For the butyrogenic CM 2 microbiota, both WBAX and RFAX induced higher metabolite production compared to the control (Figures 2C,D). Differences in the production kinetics of intermediates (formate, lactate, and succinate) and end metabolites (acetate, propionate, and butyrate) between WBAX and RFAX and among differently processed AX were observed (Figure 2 and Supplementary Figure 2). Among all tested AX, the WBAX samples had the greatest effect on metabolic activity, leading to a strong formation of butyrate at the end of the fermentation, with the highest butyrate production with extruded WBAX (Figure 3B and Supplementary Figure 2). Similar to the findings of CM 1, native WBAX showed higher succinate accumulation at 12 and 24 h compared to extruded and milled WBAX (12 h: native 23 ± 6 mM; extruded: 16.6 ± 0.4 mM; milled: 11 ± 2 mM). Compared to native and milled WBAX, extruded WBAX resulted in a higher lactate concentration at 12 h, which was metabolized at 24 and 48 h. Interestingly, extruded WBAX induced a faster and higher butyrate production by CM 2 compared to native and milled WBAX (Supplementary Figure 2). In contrast to WBAX, the RFAX fermentation resulted in lower amounts of metabolites with the accumulation of formate and lower butyrate levels (Figures 2D, 3B). Moreover, formate accumulation was higher with extruded RFAX compared to native RFAX at all timepoints (12 h: 29.9 ± 1.7 mM and 19.6 ± 1.4 mM, respectively).

In sum, the considerable variations in metabolite production between differently processed AX, especially observed for WBAX, in both CMs, indicate that the structural AX characteristics impacted the fermentation behavior.

### Changes in the Microbial Community Through Differently Processed AX Fermentation

#### Variations in Microbial Composition Between Microbiota, AX Source, and Processing Method

The microbiota after 12 and 24 h of fermentation of differently processed WBAX and RFAX were analyzed using 16S rRNA gene amplicon sequencing. Samples of standard and oxidized standard AX were not included.

**Figure 4:**
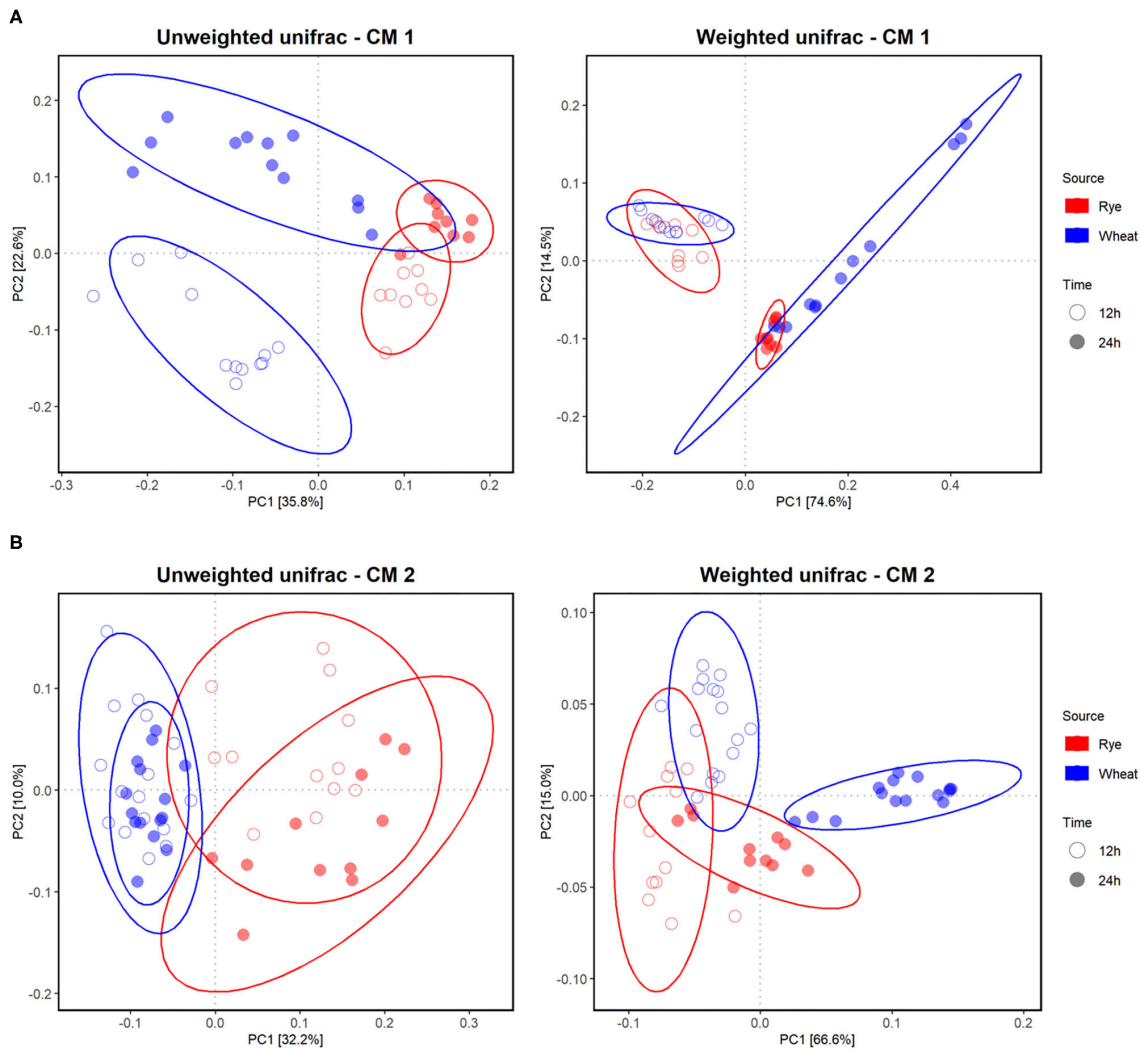
Effect of different AX sources, namely, WBAX (blue) and RFAX (red), on the *in vitro* colon microbiota composition in CM 1 **(A)** and 2 **(B)**. Principle Coordination Analysis (PCoA) of incubated microbiota based on the unweighted and weighted UniFrac analysis matrix on ASV level. Each point represents an *in vitro* colon microbiota sample after 12 (◦) or 24 h (•) of fermentation. Ellipses indicate 95% confidence intervals.

**Figure 5:**
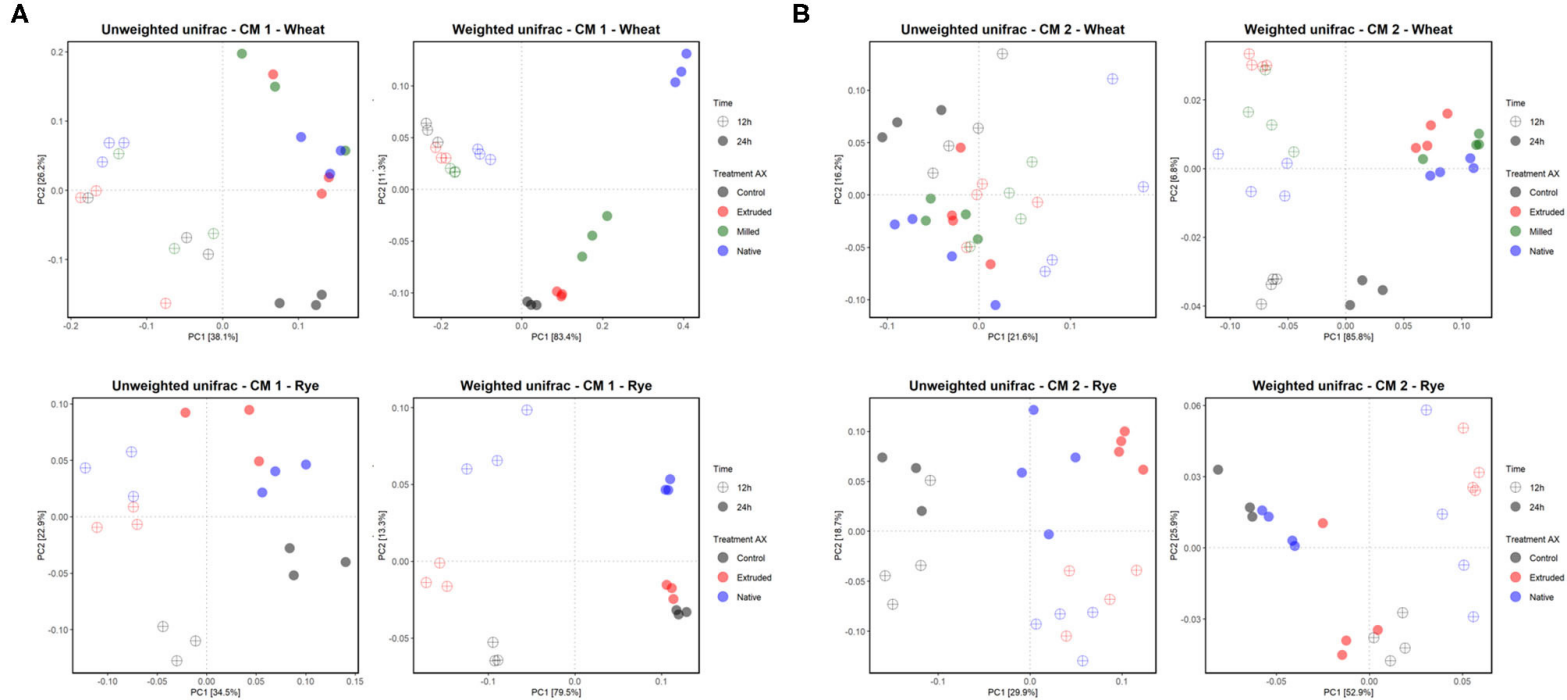
Effect of differently processed AX namely native, extruded, and milled WBAX native and extruded RFAX on the microbiota composition of CM 1 **(A)** and CM 2 **(B)**, respectively. PCoA of incubated microbiota based on unweighted and weighted UniFrac analysis matrix on ASV level. Each point represents an *in vitro* colon microbiota sample after 12 (◦) or 24 h (•) of fermentation and samples are colored based on treatment with control non-AX supplemented microbiota.

The bacterial alpha diversity between the differently processed AX treatments was comparable within each microbiota after *in vitro* fermentation (Supplementary Figure 4). The beta diversity, which gives the variation in microbiota composition between samples, was evaluated to reveal differences of the microbial community between CMs, AX sources, and between differently processed AX. In general, the *in vitro* colon microbiota composition differed between CMs as indicated in a principle coordination analysis (PCoA)-biplot on weighted and unweighted UniFrac distance (Supplementary Figure 5). Furthermore, clear differences in the *in vitro* microbiota composition between RFAX and WBAX fermentation within both CMs were observed in unweighted and weighted UniFrac PCoA-biplots (Figure 4). The PERMANOVA analysis did not detect differences between differential processed AX for the unweighted UniFrac metrics except for CM 2, where extruded RFAX samples differed from native RFAX at 24 h (*p* < 0.05; Figure 5). When considering the ASV abundance, which is given by the weighted UniFrac distance, the PERMANOVA analysis detected differences between differently processed WBAX and RFAX at 12 and 24 h in both CM 1 (*p* < 0.1) and CM 2 (*p* < 0.05; Figure 5). In the case of CM 1, WBAX showed distinct clusters depending on the treatment after 24 h of fermentation, whereas RFAX exhibited strong variations after 12 h (Figures 5A,B). The microbiota of CM 2 showed differences after 12 and 24 h for the fermentation of differently processed WBAX and RFAX (Figures 5C,D). These assembled data suggest that the microbial composition and, in particular, the abundance of detected bacterial taxa depends on the AX source and the processing method indicating the influence of structural characteristics.

**Figure 6:**
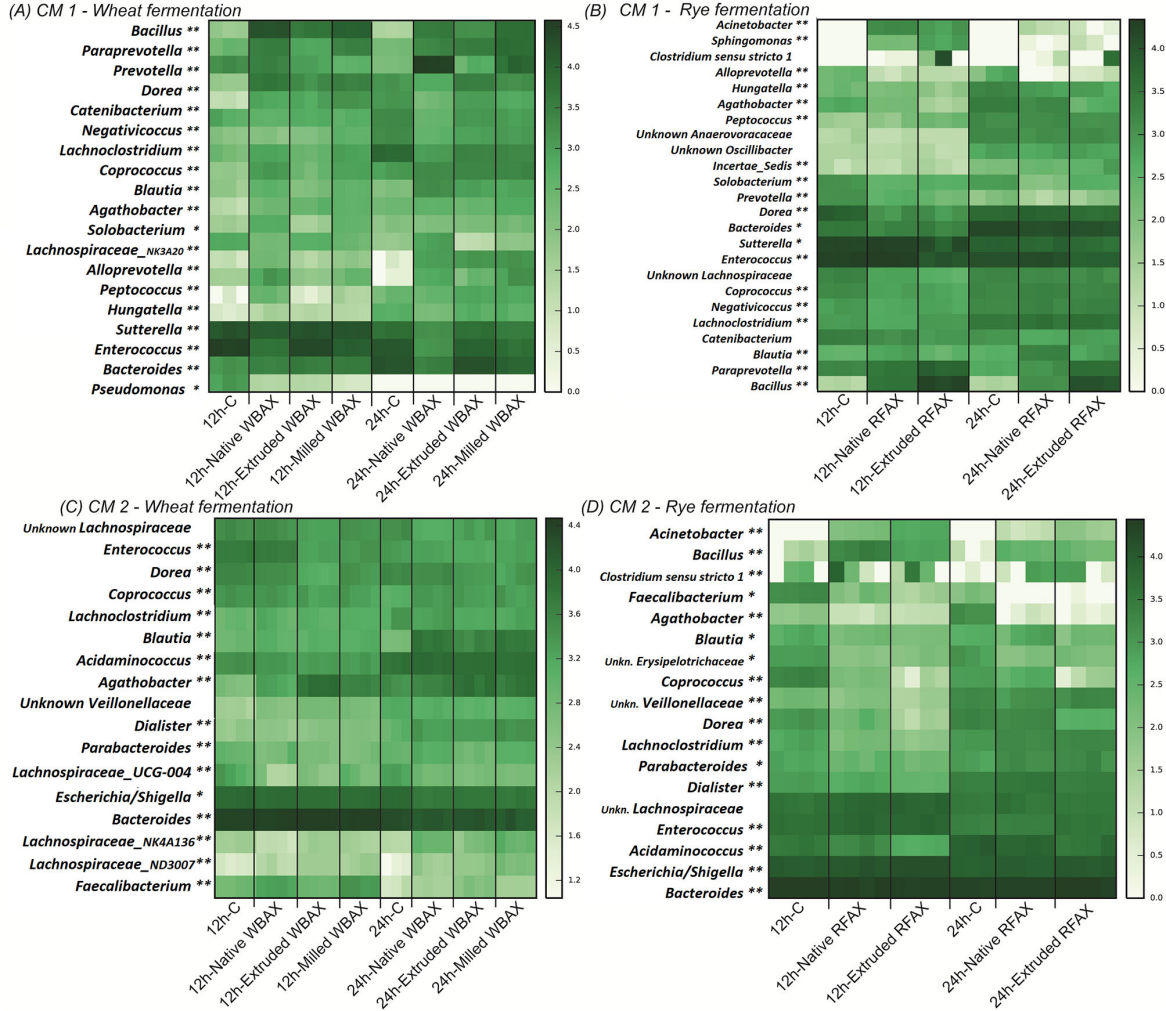
Heatmap representing log-transformed absolute genus abundance (>1%) identified in CM 1 and CM 2 after 12 or 24 h of fermentation of differently processed WBAX **(A,C)** and RFAX **(B,D)**, respectively. Genera for which abundance was significantly different between control and AX treatments at 12 and 24 h (**) or only at 12 (*) or 24 h (*) are indicated. Specific outcome of DESeq analysis is summarized in Supplementary Tables 7–10.

Consistently, the evaluation of abundant bacterial genera in the community revealed distinct differences between AX sources in their relative abundance (Figure 6 and Supplementary Figure 6; Supplementary Table 10). In both microbiota, after 24 h of fermentation, WBAX resulted in a higher abundance of *Blautia* and decreased abundance of *Enterococcus* and *Bacteroides* compared to control. In contrast, RFAX specifically enhanced the abundance of *Bacillus* and *Acinetobacter*, while lowering the abundance of *Agathobacter* when compared to control. Besides general effects on the microbial composition observed in both CMs, AX source also induced microbiota specific changes. The WBAX, for example, led in CM 1 to strong increases in abundance of *Bacillus, Paraprevotella*, and *Alloprevotella*, while in CM 2, it resulted in the increased abundance of *Faecalibacterium* and *Lachnospiraceae* genera compared to control (|Log2FC| > 3, Supplementary Tables 7, 9).

The comparison between the differently processed WBAX and RFAX exhibited stronger Log2FoldChanges in genera present in CM 1 than in CM 2. Compared to native WBAX, processed WBAX resulted in CM 1 in lower abundances of the *Prevotella* and a *Lachnospiraceae* genera and higher abundance of *Enterococcus* and *Bacteroides* (Figure 6A and Supplementary Table 7). Type of WBAX processing also had an impact on the microbiota composition after fermentation. In CM 1, extruded WBAX led to a lower abundance of *Prevotella, Alloprevotella*, and *Lachnospiraceae* genera compared to milled WBAX. Interestingly, the abundance of *Faecalibacterium* in CM 2 with extruded WBAX was lower after 12 h of fermentation but higher after 24 h of fermentation when compared to native and milled WBAX (Figure 6C and Supplementary Table 9). The compositional effects of RFAX processing were mainly detected after extrusion. Compared to native RFAX, extruded RFAX resulted in higher abundances of *Alloprevotella, Bacillus*, and *Solobacterium* and lower abundances of *Agathobacter* in CM 1 (Figure 6B and Supplementary Table 8). While in CM 2, extruded RFAX led to lower abundances of *Coprococcus, Dorea*, and *Blautia* compared to native RFAX (Figure 6D and Supplementary Table 10).

#### Differences in Total Bacteria, *Lactobacillus/Leuconostoc/Pediococcus* and *Bifidobacteria* Concentrations Associated With Microbiota, AX Source, and Processing Method

**Figure 7:**
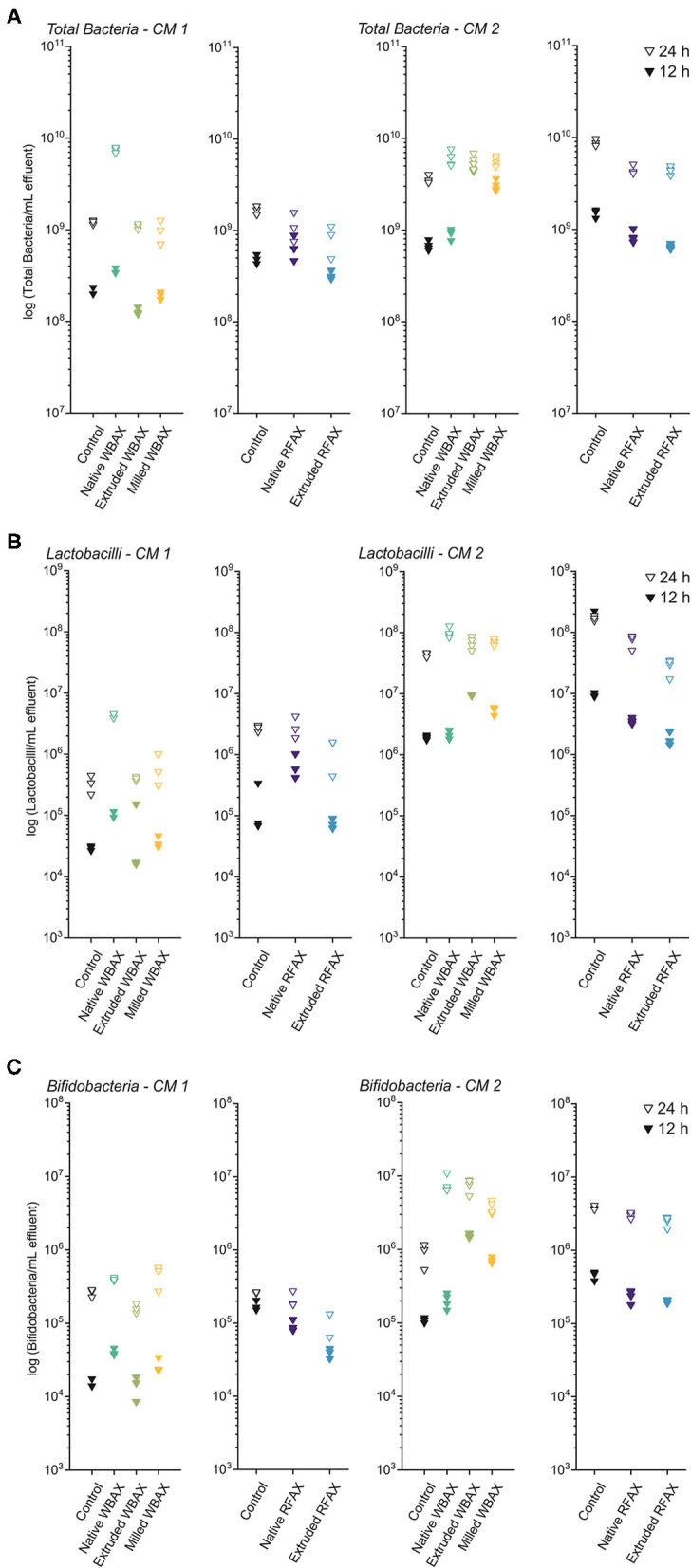
Bacterial quantification by quantitative PCR targeting total bacteria **(A)**, *Lactobacillus/Leuconostoc/Pediococcus* spp. (*LLP*) **(B)**, and *Bifidobacterium* spp. **(C)** at 12 and 24 h of *in vitro* colon fermentation of differently processed WBAX and RFAX by two CM 1 and CM 2.

Quantitative PCR targeting total bacteria, *Lactobacillus*/*Leuconostoc*/*Pediococcus* spp. (*LLP*) and *Bifidobacteria* spp. was performed to assess the microbial growth after 12 and 24 h of AX fermentation in the differently processed WBAX and RFAX extracts (Figure 7).

In both microbiota, total bacterial growth was observed between 12 and 24 h of fermentation of AX, with higher end bacterial concentrations in CM 2 compared to CM 1 (Figure 7A). For both timepoints and CMs, RFAX did not promote the total bacterial growth compared to the control fermentation. Cultivated microbiota 1 (CM1) showed faster and higher growth with native WBAX (12 h: 3.5 × 10^8^ ± 2.5 × 10^7^ bacteria/mL) compared to extruded (12 h: 1.3 × 10^8^ ± 1.2 × 10^7^ bacteria/mL) and milled (1.9 × 10^8^ ± 1.7 × 10^7^ bacteria/mL) WBAX. While CM 2, in contrast, showed faster bacterial growth with extruded and milled WBAX (12 h: 4.7 × 10^9^ ± 4.0 × 10^8^ and 3.1 × 10^9^ ± 4.1 × 10^8^ bacteria/ml, respectively) compared to native WBAX (12 h: 9.2 × 10^8^ ± 1.1 × 10^8^ bacteria/ml).

Native WBAX resulted in both microbiota in higher end concentrations of *LLP* compared to control fermentations (24 h, CM 1: 4.4 × 10^6^ ± 3.8 × 10^5^ vs. 3.4 × 10^5^ ± 1.1 × 10^5^ bacteria/ml, CM 2: 1.1 × 10^8^ ± 2.0 × 10^7^ vs. 4.4 × 10^7^ ± 3.7 × 10^6^ bacteria/ml) (Figure 7B). In contrast, processed WBAX resulted in lower *LLP* concentrations in CM 1 at 12 h fermentation, but higher *LLP* concentrations in CM 2 compared to native WBAX. RFAX did not promote the growth of *LLP* compared to the control fermentation and even resulted in lower end concentrations in CM 2, especially for extruded RFAX (24 h: 3.3 × 10^7^ ± 2.5 × 10^6^ vs. 1.7 × 10^8^ ± 1.7 × 10^7^ bacteria/ml) (Figure 7B).

The growth of *Bifidobacteria* spp. was stimulated by differently processed WBAX compared to control fermentation, especially in CM 2 resulting in higher *Bifidobacteria* concentrations compared to CM 1 after WBAX fermentation (24 h, CM 1: 4 × 10^5^ ± 2.1 × 10^4^ vs. CM 2: 7.8 × 10^6^ ± 2.2 × 10^6^ bacteria/ml) (Figure 7C). Compared to native WBAX, the extruded and milled WBAX induced a faster *Bifidobacteria* growth in CM 2 microbiota (12 h: native: 2 × 10^5^ ± 4.7 × 10^4^; extruded: 1.6 × 10^6^ ± 9.8 × 10^4^; milled: 7.2 × 10^5^ ± 5.9 × 10^4^ bacteria/ml) but not in CM 1 microbiota. The different RFAX samples did not enhance the growth of *Bifidobacteria* compared to the control and even resulted in lower concentrations, as in the case of the extruded RFAX in CM 1 (24 h: 1.1 × 10^5^ ± 4 × 10^4^ vs. 2.6 × 10^5^ ± 4.2 × 10^3^ bacteria/ml) (Figure 7C).

## Discussion

The objective of this study was to evaluate the effects of naturally occurring and processing-induced structural alterations of soluble AX on the *in vitro* human colon fermentation. We hypothesized that fermentability and prebiotic potential would improve with decreasing M_w_ and decreasing A/X ratio, and that these structural differences between wheat and rye AX and between differently processed AX would be reflected in SCFA production and the growth of health-associated taxa.

All differently processed WBAX and RFAX stimulated the production of SCFA, which is consistent with previous AX studies (57, 58). However, the extent of fermentation differed between AX fractions and source. RFAX showed a lower fermentability compared to WBAX. This implies that naturally given structural alterations such as the substitution pattern in wheat bran and rye flour AX affect their *in vitro* colon fermentation behavior. This was also observed for rye, wheat, and oat bran (59) or AX isolated from corn, wheat, or rice brans (25, 60). Ultimately, further studies need to focus on a detailed structural characterization of differently processed AX derived from various grains.

Native WBAX, which had the highest M_w_ and A/X ratio, showed the highest fermentability in both microbiota, while processed WBAX with lower M_w_ and a A/X ratio resulted in a lower acidification and SCFA production. These findings contradict the hypothesis of our study and previous studies, which suggest that low M_w_ improves AX fermentability because it facilitates the access of enzymes (20). Furthermore, the data show that there is no direct link between A/X ratio and the resulting fermentation behavior. This is in line with the observations made by Rumpagaporn et al. (25) who did not observe a correlation between molecular size, A/X ratio, and degree of substitution of AX and their fermentation rate by human fecal microbiota; instead, they observed that branching and high complex sidechains have a greater effect on the fermentation kinetics. Consequently, an in-depth structural investigation of linkage and substitution patterns by NMR or LC-MS, as already reported for AXOS, would be required to identify the governing structural features influencing the prebiotic potential of the differently processed WBAX and RFAX (61, 62).

Our results suggest that the amount of bound phenolic acids in AX, which were much higher in RFAX and increased by milling and extrusion in both cereals, may inhibit and slow down the AX fermentation. It was hypothesized by Snelders et al. (62) that bound phenolic acids such as ferulic acids may cause sterical effects, limiting the accessibility for AX degrading enzymes and consequently inhibiting AX fermentation. Moreover, the authors reported that the antimicrobial properties of feruloylation inhibited AXOS fermentation, which may also explain the overall lower fermentability of the RFAX compared to WBAX observed in this study. Interestingly, significant differences in SCFA production between AX treatments disappeared after 48 h, despite higher bound phenolic acid content, implying that any initial effect of bound phenolic acid content did not persist. This could be because, once the steric hindrance had been cleaved by ferulolyl esterase-producing bacteria, the extruded WBAX was more degradable due to its less dense structure.

The fermentation rate of WBAX differed between the *Prevotellaceae-* (CM 1) and the *Bacteroidaceae-* (CM 2) dominated microbiota. The CM 2 microbiota showed a stronger bifidogenic and butyrogenic response upon WBAX compared to CM 1, which is in line with previous findings *in vivo* (22, 63). This can be the result of cross-feeding reactions between bifidobacteria- and butyrate-producing WBAX responding to *Faecalibacterium* and *Lachnospiraceae* taxa (23, 64). In CM 1, WBAX promoted propionate production and the growth of *Prevotella*, which corresponds with a recent study in healthy elderly that identified the *Prevotella* genus as a driving factor in microbiota response to wheat bran AX (65). The bloom of *Prevotella* also explained the succinate accumulation especially observed in native WBAX in CM 1, as some *Prevotella* species appear to produce succinate preferentially over propionate (66).

The extrusion of WBAX enhanced the butyrate production and abundance of *Faecalibacterium*, a genus associated with health (67) in butyrogenic CM 2 but did not impact fermentation in CM 1. This may be because of the low abundance of *Faecalibacterium* in CM 1 and the observed growth inhibition of its main AX fermenting genus, *Prevotella*, during extruded WBAX fermentation. Also, during milled WBAX fermentation, a lower succinate production combined with a decreased abundance of *Prevotella* was observed. The lower fermentability of processed WBAX by propiogenic CM 1 may be explained by their higher levels of bound phenolic acids compared to native WBAX and their described antimicrobial activity (68). Indeed, previous *in vitro* work showed that ferulic acid-rich fiber fractions slowed down fermentation and, subsequently, inhibited propionate production (69, 70) or resulted in more butyrogenic fermentation (71). Hence, the identified differences in abundant taxa and end metabolites highlight the strong influence of AX processing on the microbial community and fermentation.

One limitation of our work is that we did not incorporate an *in vitro* upper digestive tract pre-digestion of the AX extracts prior to *in vitro* colonic fermentation. This was to avoid the potential loss of water-extractable AX during such *in vitro* pre-digestions (16, 28), which would complicate the comparison of the fermentation behavior of the differently processed AX. However, we cannot exclude that, *in vivo*, the upper digestive tract passage may impact the AX extracts and their consecutive colonic fermentation. For instance, it was demonstrated that hydrochloric acid treatments lead to the degradation of arabinose substitutions within the AX chains (36). Therefore, future *in vitro* studies could investigate if processing-induced changes in soluble AX and their prebiotic potential are preserved after upper digestive tract conditions. Another limitation of the study is that only two microbiota were studied. Although common AX-induced effects on fermentation behavior were observed, several specific effects such as type of SCFA and type of bacterial taxa promoted differed between the microbiota. Several other *in vitro* studies with other types of wheat bran or AX extracts observed this individual microbiota response as well (26, 69, 72). In addition, data from human intervention studies often show very individual responses in microbiota change upon AX supplementation (24, 65), and their outcome is hard to predict due to the complex cross-feeding and ecological interactions occurring within the microbiota. The current setup is, therefore, a first step in gaining knowledge on the impact of processing-induced changes in soluble AX on fermentation kinetics and affected bacterial taxa but, obviously, there is a need for further investigation and validation with more individual microbiota *in vivo*. Such studies may allow for the selection of processing methods for producing AX fractions with different structural features and with predictable prebiotic shifts in gut microbiota (73).

## Conclusion and Outlook

Prior research has often presented AX as a promising food constituent to prevent particular diet-related chronic diseases, which is associated with many beneficial effects resulting from its prebiotic activity. This study is the first report investigating the structure–function relation between naturally occurring and processing-induced structural alterations in soluble AX and its effect on *in vitro* colon fermentation.

The results demonstrate that fermentation behavior is strongly linked to the AX fine structure and their processing-induced modifications. The SCFA metabolism, acidification kinetics, bacterial growth, and bacterial composition revealed that wheat bran AX was fermented faster than rye flour AX. Extruded or milled AX did not enhance fermentation, nor did AX isolates with lower M_w_ and A/X ratio. High levels of bound phenolic acids were identified as an inhibiting factor for AX fermentation kinetics. Bacterial genera promoted by AX varied between AX source and processing type, but also between microbiota. Extruded WBAX promoted butyrate production and the growth of butyrate-producing *Faecalibacterium* in the butyrogenic microbiota but did not enhance fermentation in the propiogenic microbiota. These inter-individual differences highlight the particular importance to elucidate further individual responses to differently processed AX in future studies. Further investigations focusing on the effect of processing-induced AX sugar functionalization or comprehensive linkage analysis are recommended, as well. This work provides the scientific framework for future *in vitro* fermentation and *in vivo* studies with processed dietary fibers.

## Data Availability Statement

The datasets presented in this study can be found in online repositories. The names of the repository/repositories and accession number(s) can be found below: https://www.ebi.ac.uk/ena, PRJEB44740.

## Author Contributions

TD, LN, and AG conceived the experiments. TD and VE conducted AX experiments and LB provided PolyFermS microbiota and data. TD, VE, and AG analyzed data and prepared figures. TD and AG wrote the manuscript and all authors reviewed the manuscript.

## Conflict of Interest

The authors declare that the research was conducted in the absence of any commercial or financial relationships that could be construed as a potential conflict of interest.

## Publisher's Note

All claims expressed in this article are solely those of the authors and do not necessarily represent those of their affiliated organizations, or those of the publisher, the editors and the reviewers. Any product that may be evaluated in this article, or claim that may be made by its manufacturer, is not guaranteed or endorsed by the publisher.
