# Supplementary material for: In vitro Colon Fermentation of Soluble Arabinoxylan Is Modified Through Milling and Extrusion
Source: Front Nutr. 2021 Aug 25;8:707763. doi: 10.3389/fnut.2021.707763 (PMC8424098; doi:10.3389/fnut.2021.707763)
Supplement: Supplementary file 1 [file Data_Sheet_1.pdf]

Supplementary Material

Supplementary S1: *In vitro* proximal colon microbiota composition of CM 1 and CM 2 and corresponding donor fecal microbiota FM 1 and FM 2 on phylum level and most abundant families (>0.5%) obtained by V4 region 16S amplicon sequencing.

	**FM 1**	**CM 1**	**FM 2**	**CM 2**
Bacteroidota	61.68%	57.08%	27.13 %	39.51 %
Firmicutes	35.68 %	37.42 %	66.17 %	50.69 %
Proteobacteria	0.30 %	4.10 %	0.17 %	9.11 %
Actinobacteria	0.87 %	0.44 %	4.58 %	0.62 %
Euryarchaeota	0.79 %	0.22 %	0.00 %	0.00 %
Desulfobacterota	0.00 %	0.00 %	0.49 %	0.00 %
Verrucomicrobiota	0.51 %	0.00 %	0.76 %	0.00 %
Cyanobacteria	0.15 %	0.73 %	0.70 %	0.00 %

**Family**	FM 1	CM 1
Prevotellaceae	54.30 %	56.39 %
Lachnospiraceae	11.61 %	16.66 %
Ruminococcaceae	3.61 %	9.38 %
Enterococcaceae	0.00 %	5.02 %
Oscillospiraceae	8.14 %	3.74 %
Veillonellaceae	0.89 %	2.17 %
Sutterellaceae	0.17 %	2.15 %
Pseudomonadaceae	0.00 %	1.95 %
Muribaculaceae	1.56 %	0.54 %

**Family**	FM 2	CM 2
Bacteroidaceae	24.51 %	39.30 %
Lachnospiraceae	41.71 %	22.31 %
Ruminococcaceae	14.71 %	14.05 %
Acidaminococcaceae	0.12 %	8.84 %
Enterobacteriaceae	0.11 %	5.22 %
Veillonellaceae	0.65 %	2.27 %
Xanthomonadaceae	0.00 %	2.03 %
Enterococcaceae	0.01 %	1.71 %
Pseudomonadaceae	0.02 %	1.26 %
Bacillaceae	0.01 %	0.61 %
Coriobacteriaceae	0.99 %	0.60 %
Moraxellaceae	0.00 %	0.58 %

Supplementary S2: Noteworthy butyrate and propionate formation through differently processed soluble WBAX and RFAX. Note that the RFAX fermentation of CM 1 did not show considerable differences in the propionate or butyrate formation and is therefore not shown.

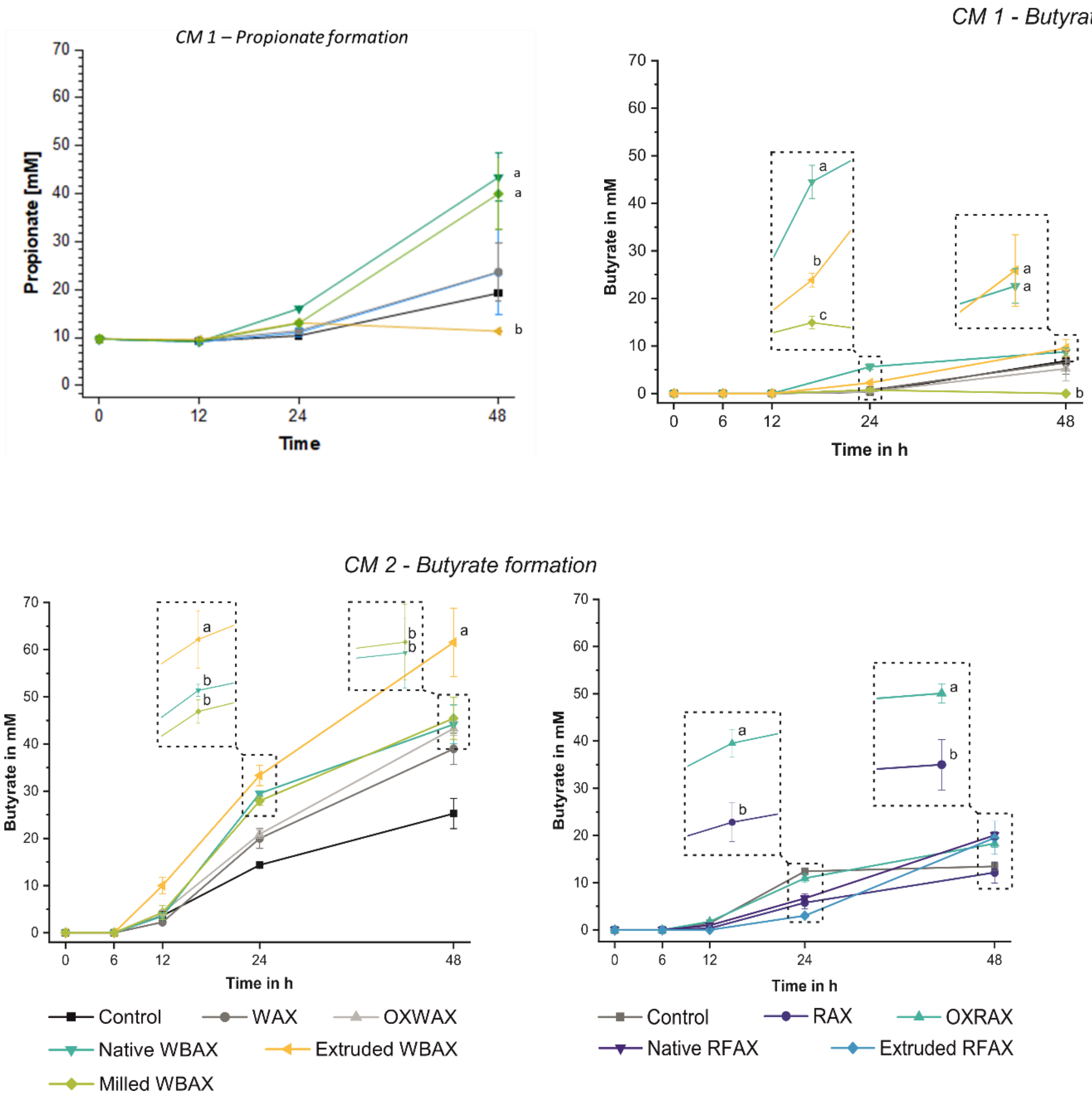

Supplementary S3: Microbial BCFA kinetics and composition (n ≥ 4) formed through the *in vitro* fermentation for 48 h of differently processed soluble WBAX and RFAX by CM1 and CM2.

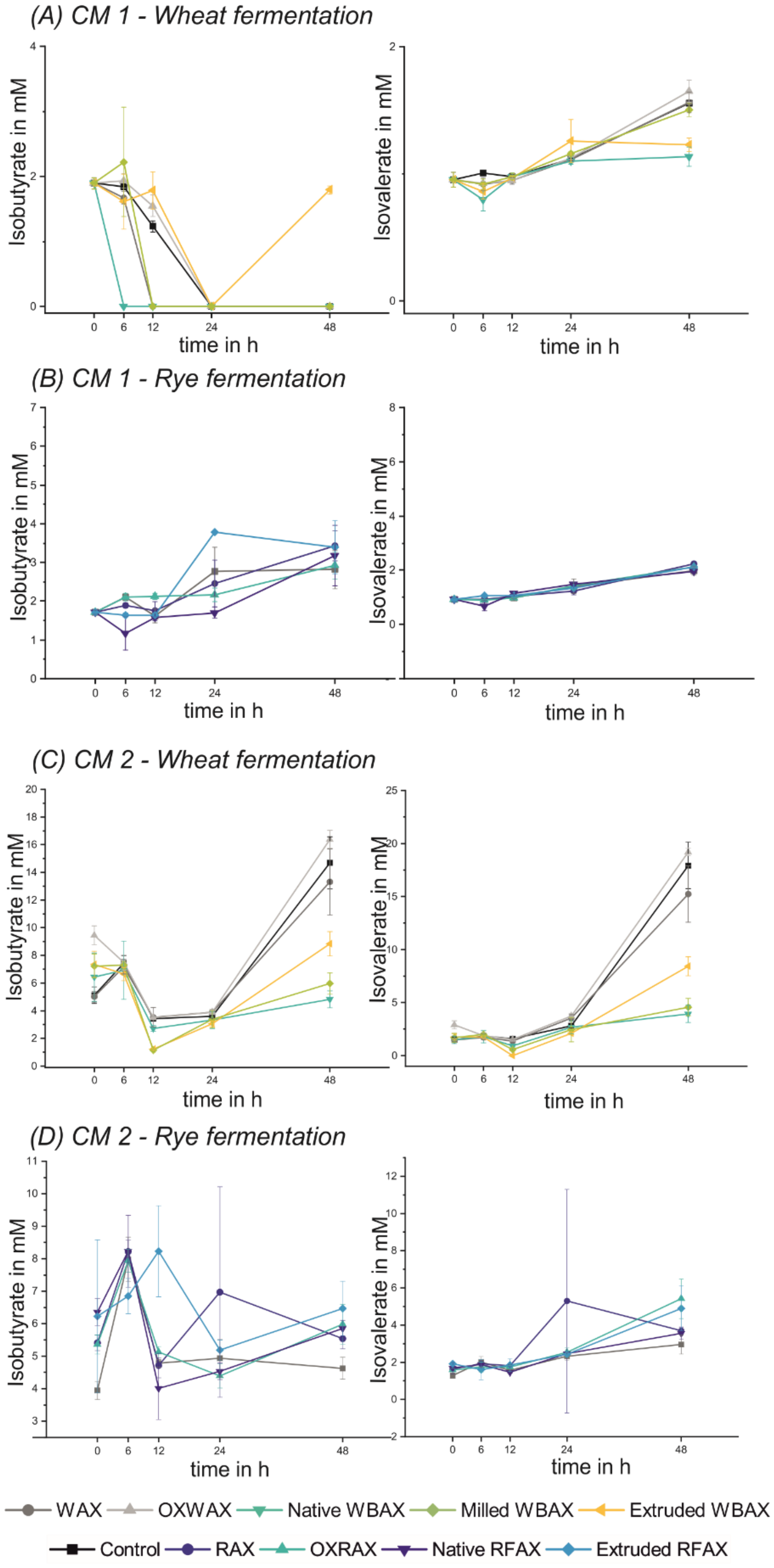

Supplementary S4: Alpha diversity expressed as richness of CM 1 (A) and CM 2 (B) after 12 h and 24 h fermentation with differently processed WBAX and RFAX. Pairwise comparison with Kruskal-Wallis test did not detect significant differences between differently processed AX.

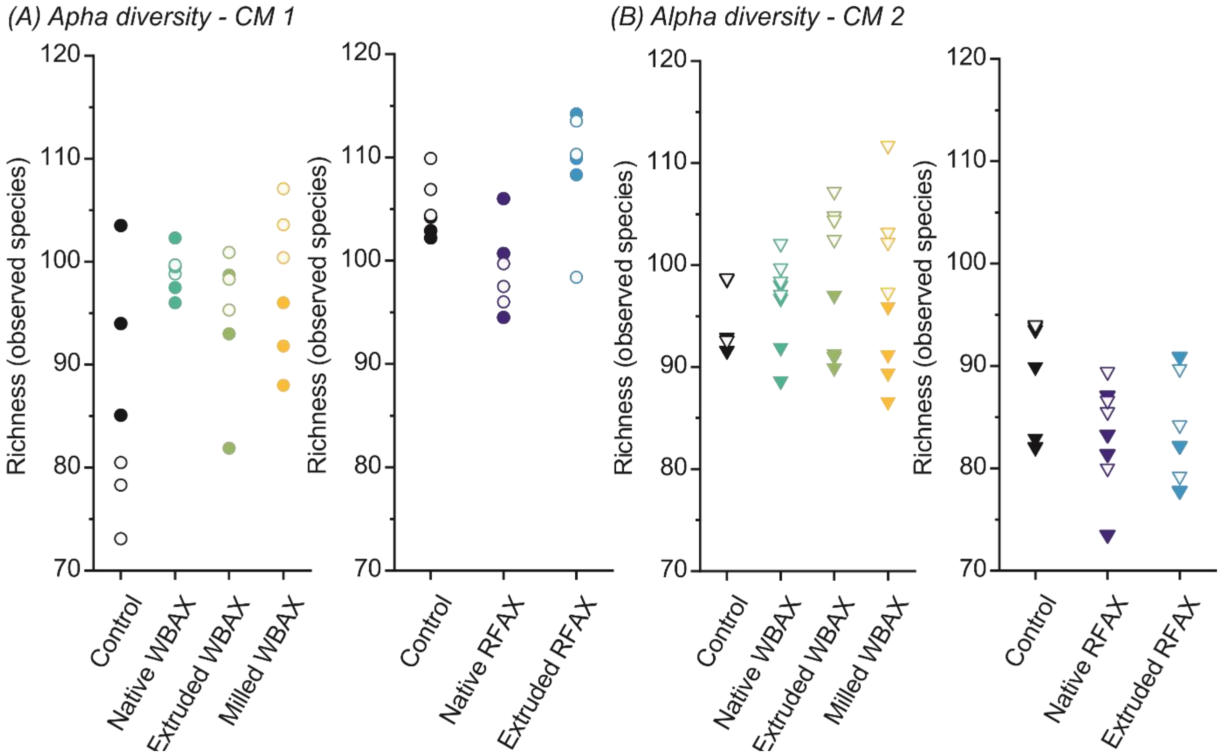

Supplementary S5: Variation in the *in vitro* microbiota composition between both CMs.

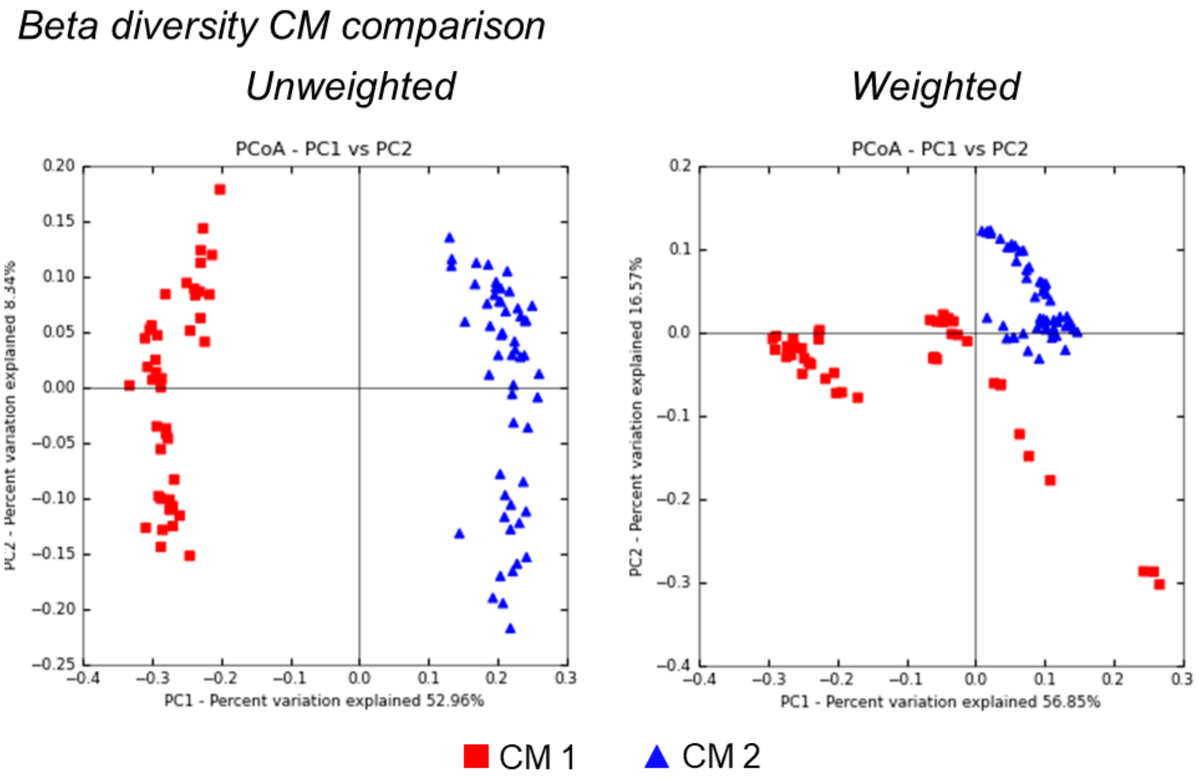

Supplementary S6: Relative abundance of taxa on family level identified in CM 1 and CM 2 after 12 h and 24 h fermentation of differently processed WBAX and RFAX.

CM 1 – Wheat fermentation

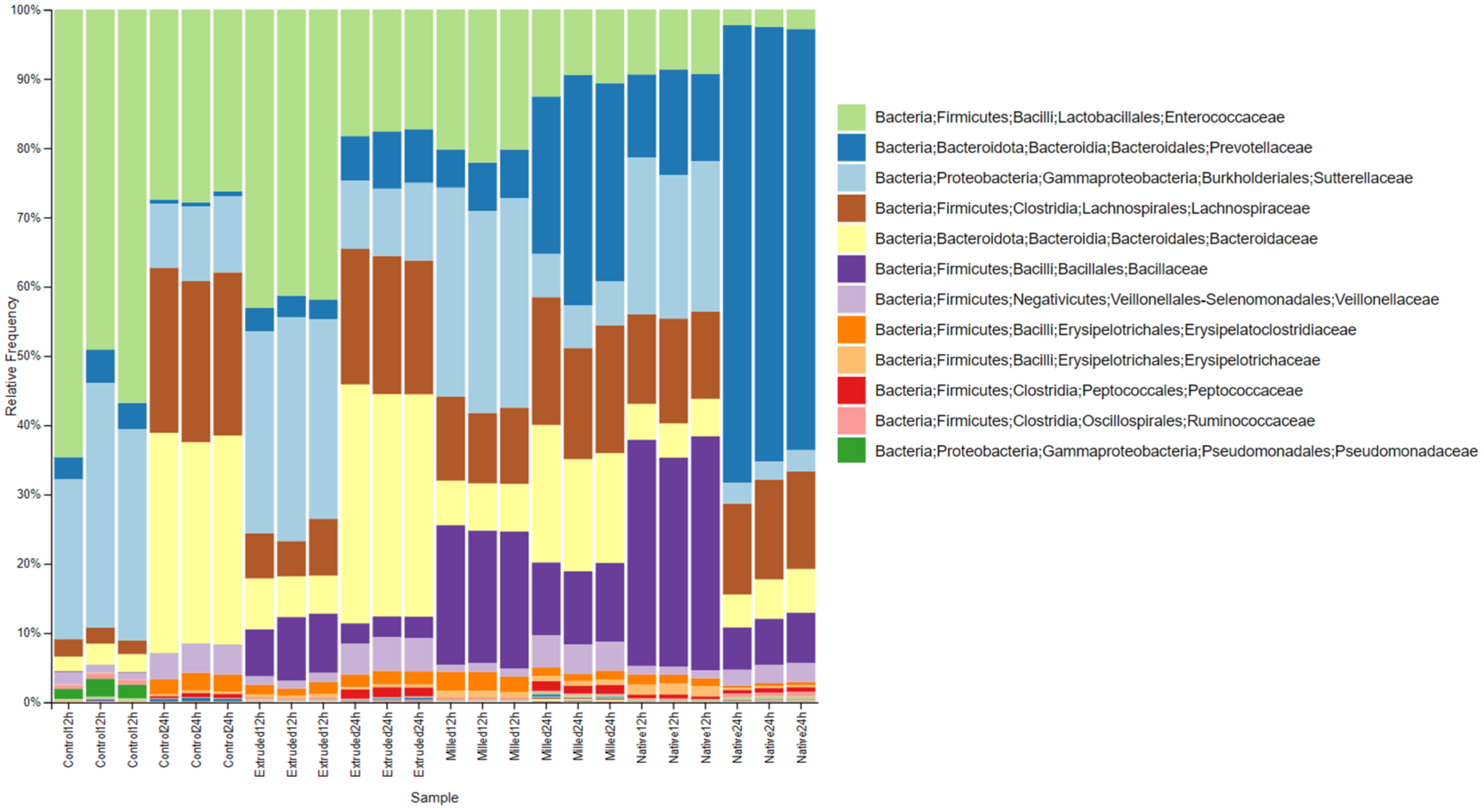

CM 1 – Rye fermentation

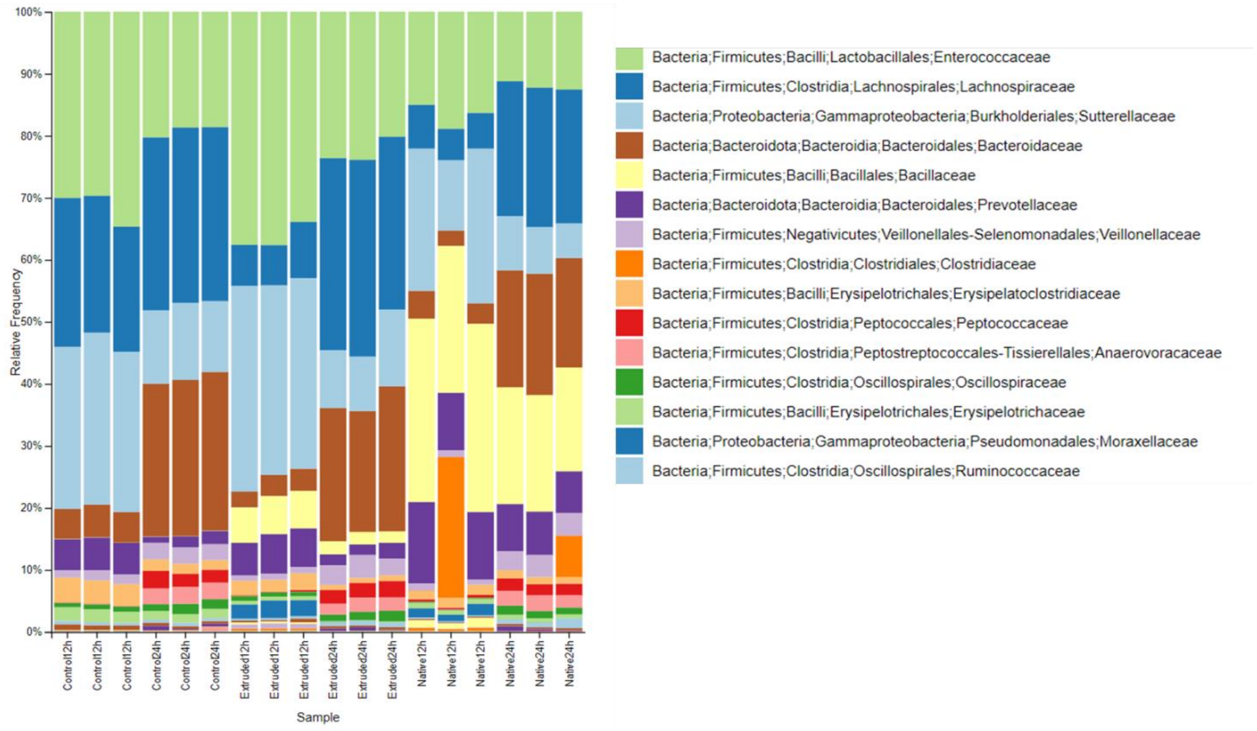

CM 2 – Wheat fermentation

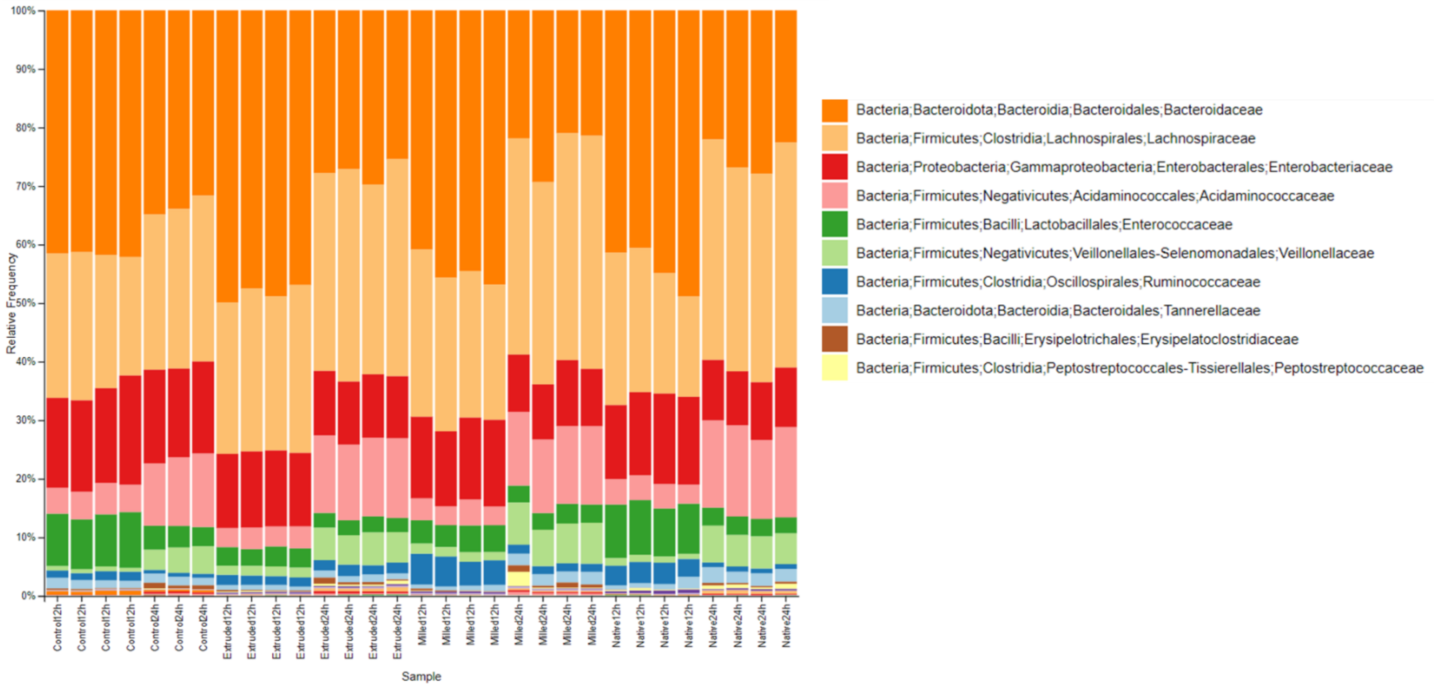

CM 2 – Rye fermentation

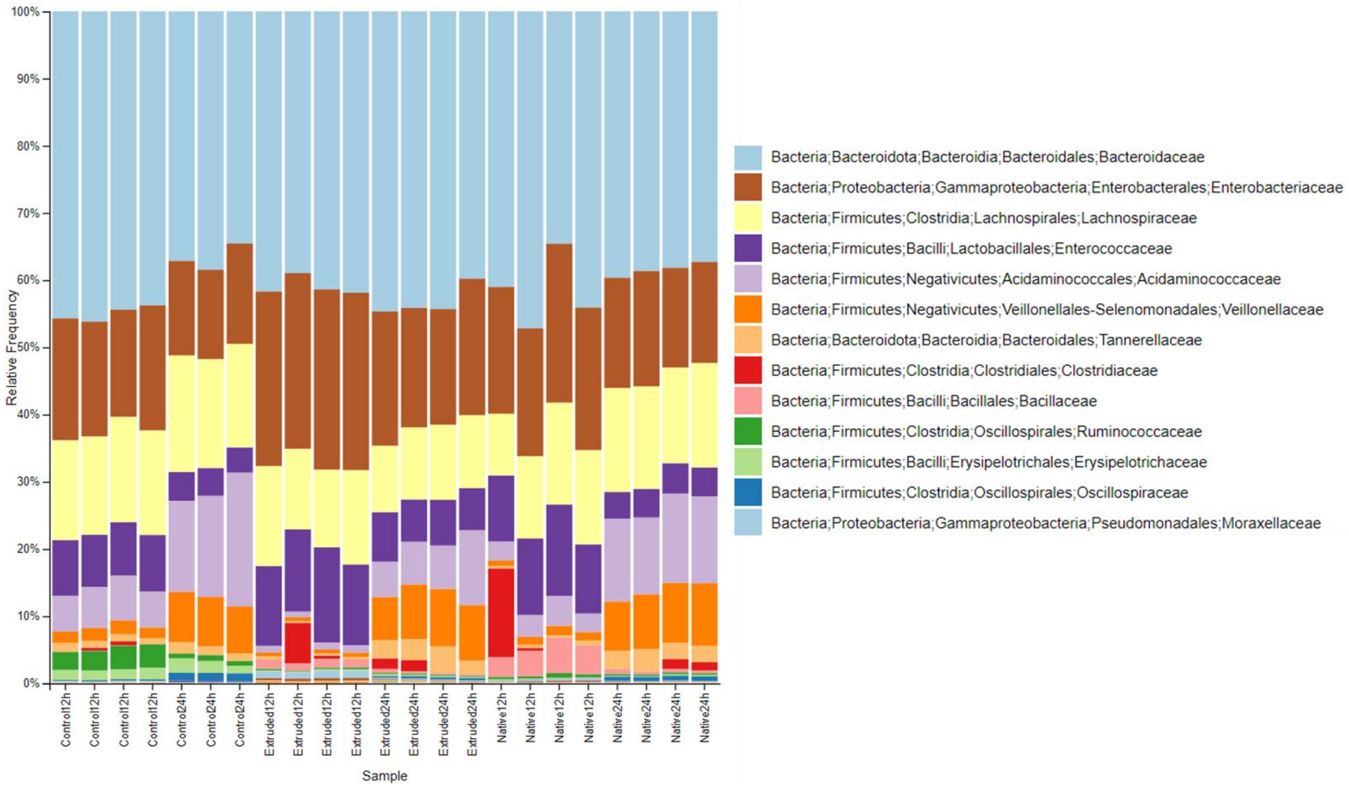

Supplementary S7: WBAX results in differences in microbial composition at genus level in **CM 1** after 12 h and 24 h fermentation *in vitro*. Table with the Log2FoldChange for pairwise significantly abundant genera ($p < 0.05$) as output from DESeq analysis. The order of listed genera corresponds to order in Figure 6A. ns=non significant

CM 1		Control vs.			Native vs.		Extruded vs.
	**Genus**	**Native**	**Extruded**	**Milled**	**Milled**	**Extruded**	**Milled**
12 h	***Bacillus***	-7.68	-6.22	-7.38	ns	1.85	1.16
	Paraprevotella	-2.86	-1.20	-3.06	-0.60	ns	1.86
	Prevotella	ns	1.22	2.43	2.78	6.85	1.21
	Dorea	-5.42	-4.77	-5.32	-0.63	-1.55	0.54
	Catenibacterium	-5.31	-5.89	-6.67	-0.83	-1.56	0.78
	Negativicoccus	1.37	ns	0.54	-0.91	-1.34	ns
	Lachnoclostridium	-1.12	ns	-1.74	ns	-0.63	1.39
	Coprococcus	-3.56	-1.96	-3.50	0.45	0.43	1.54
	Blautia	-2.11	-1.13	-2.46	1.99	1.75	1.33
	Agathobacter	-3.04	-4.18	-4.26	ns	1.17	ns
	Solobacterium	-3.20	ns	-2.91	ns	0.43	3.08
	Lachnospiraceae_NK3A20_group	2.41	0.70	1.62	4.25	6.51	-0.92
	Alloprevotella	-3.97	ns	-2.51	ns	2.12	1.98
	Peptococcus	-6.27	ns	-3.48	ns	-0.49	2.33
	Hungatella	-1.95	-2.01	-2.07	-0.66	-0.85	ns
	Sutterella	1.19	ns	ns	-0.32	-1.11	ns
	Enterococcus	3.39	0.67	1.79	-1.33	-2.11	-1.12
	Bacteroides	ns	-1.05	-1.04	-0.85	-1.85	ns
	Pseudomonas	6.42	6.13	7.91	ns	ns	-1.78
24 h	***Bacillus***	-6.90	-5.05	-6.83	ns	1.85	1.78
	Paraprevotella	-3.39	-3.18	-3.99	-0.60	ns	0.82
	Prevotella	-7.48	ns	-4.70	2.78	6.85	4.08
	Dorea	1.30	ns	0.66	-0.63	-1.55	-0.92
	Catenibacterium	2.85	1.28	2.02	-0.83	-1.56	-0.74
	Negativicoccus	3.05	1.71	2.14	-0.91	-1.34	-0.43
	Lachnoclostridium	3.13	2.50	2.78	ns	-0.63	ns
	Coprococcus	-0.67	-0.24	ns	0.45	0.43	ns
	Blautia	-3.57	-1.82	-1.58	1.99	1.75	ns
	Agathobacter	-0.48	0.69	-0.33	ns	1.17	1.02
	Solobacterium	ns	ns	ns	ns	0.43	0.43
	Lachnospiraceae_NK3A20_group	-3.67	2.84	0.58	4.25	6.51	2.26
	Alloprevotella	-7.37	-5.25	-7.70	ns	2.12	2.45
	Peptococcus	ns	-0.61	-0.44	ns	-0.49	ns
	Hungatella	1.89	1.05	1.23	-0.66	-0.85	ns
	Sutterella	2.05	0.94	1.73	-0.32	-1.11	-0.79
	Enterococcus	3.68	1.57	2.35	-1.33	-2.11	-0.78
	Bacteroides	2.70	0.85	1.85	-0.85	-1.85	-1.00
	Pseudomonas	ns	ns	ns	ns	ns	ns

Supplementary S8: RFAX results in differences in microbial composition at genus level in **CM1** after 12 h and 24 h fermentation *in vitro*. Table with the Log2FoldChange for pairwise significantly abundant genera ($p < 0.05$) as output from DESeq analysis. The order of listed genera corresponds to order shown in Figure 6B. ns= non significant

CM 1		Control vs.		Native vs.
	**Genus**	**Native**	**Extruded**	**Extruded**
12 h	***Acinetobacter***	-13.57	-13.99	ns
	Sphingomonas	-12.32	-9.85	-2.47
	Clostridium sensu stricto 1	ns	ns	ns
	Alloprevotella	2.73	3.41	ns
	Hungatella	0.75	ns	0.65
	Agathobacter	2.98	1.03	1.95
	Peptococcus	-3.37	-1.63	-1.74
	Incertae_Sedis	-1.30	ns	ns
	Solobacterium	0.42	1.27	-0.85
	Prevotella	1.61	1.04	0.57
	Dorea	1.85	2.06	ns
	Bacteroides	-0.38	ns	-0.50
	Sutterella	-0.50	-0.81	ns
	Enterococcus	ns	-0.77	0.69
	Coprococcus	0.42	0.58	ns
	Negativicoccus	-0.49	ns	-0.56
	Lachnoclostridium	-1.03	ns	-1.15
	Blautia	-1.01	-1.69	0.68
	Paraprevotella	-2.86	-1.44	-1.42
	Bacillus	-10.51	-7.88	-2.63
24 h	***Acinetobacter***	-5.29	-7.67	ns
	Clostridium sensu stricto 1	ns	ns	ns
	Alloprevotella	4.87	ns	-3.52
	Hungatella	1.06	0.95	ns
	Agathobacter	2.39	8.39	2.05
	Peptococcus	ns	-0.36	ns
	Incertae_Sedis	-2.42	-1.23	-1.19
	Solobacterium	0.66	2.90	-2.24
	Prevotella	ns	1.42	ns
	Dorea	ns	-0.57	0.57
	Bacteroides	ns	ns	0.17
	Sutterella	ns	ns	0.45
	Enterococcus	0.33	-0.55	0.88
	Coprococcus	-0.40	-0.52	ns
	Negativicoccus	-0.75	-0.62	ns
	Lachnoclostridium	-0.79	-0.57	ns
	Catenibacterium	ns	0.69	-0.53
	Blautia	-1.29	-2.79	1.50
	Paraprevotella	-3.47	-1.61	-1.86
	Bacillus	-9.28	-6.05	3.23

Supplementary S9: WBAX results in differences in microbial composition at genus level in **CM 2** after 12 h and 24 h fermentation *in vitro*. Table with the Log2FoldChange for pairwise significantly abundant genera ($p < 0.05$) as output from DESeq analysis. The order of listed genera corresponds to order shown in Figure 6C. ns= non significant

CM 2	Genus	Control vs.			Native vs.		Extruded vs.
		Native	Extruded	Milled	Milled	Extruded	Milled
12 h	***Unknown Lachnospiraceae***	ns	ns	ns	ns	ns	ns
	Enterococcus	ns	1.20	1.06	1.14	1.29	ns
	Dorea	0.26	1.32	0.63	0.37	1.06	-0.69
	Coprococcus	ns	ns	0.39	ns	ns	ns
	Lachnoclostridium	-0.62	-0.81	-0.70	ns	ns	ns
	Blautia	-0.67	-0.94	-0.83	ns	ns	ns
	Acidaminococcus	ns	ns	0.37	ns	ns	ns
	Agathobacter	-1.96	-4.32	-3.20	-1.24	-2.36	1.12
	Dialister	ns	-0.72	-0.73	-0.49	-0.48	ns
	Parabacteroides	ns	ns	0.83	ns	ns	ns
	Lachnospiraceae_UCG-004	2.88	ns	1.22	-1.66	ns	ns
	Escherichia/Shigella	ns	ns	ns	ns	ns	ns
	Bacteroides	ns	-0.50	ns	ns	-0.30	0.38
	Lachnospiraceae_NK4A136_group	1.29	1.84	ns	-1.02	-1.46	0.44
	Lachnospiraceae_ND3007_group	-1.36	ns	ns	-0.97	-1.11	ns
	Faecalibacterium	-1.55	-0.40	-1.90	-0.35	1.14	-1.49
24 h	***Unknown Lachnospiraceae***	ns	ns	ns	ns	ns	ns
	Enterococcus	0.78	1.10	0.84	ns	0.32	ns
	Dorea	0.58	1.32	1.28	0.69	0.74	ns
	Coprococcus	-0.46	ns	ns	0.24	0.31	ns
	Lachnoclostridium	0.57	1.05	0.91	ns	ns	ns
	Blautia	-3.09	-2.30	-2.87	ns	0.78	-0.57
	Acidaminococcus	ns	0.38	0.40	0.28	0.26	ns
	Agathobacter	ns	-0.71	ns	ns	-0.65	ns
	Dialister	-0.61	-0.52	-0.96	-0.35	ns	-0.45
	Parabacteroides	ns	0.93	ns	ns	1.07	-0.82
	Lachnospiraceae_UCG-004	1.94	2.43	2.38	ns	0.49	ns
	Escherichia/Shigella	1.13	1.10	1.20	ns	ns	ns
	Bacteroides	0.90	0.86	1.07	ns	ns	ns
	Lachnospiraceae_NK4A136_group	-2.61	-1.69	-2.62	0.44	0.93	-0.94
	Lachnospiraceae_ND3007_group	-2.75	-3.07	-3.95	-1.19	ns	-0.87
	Faecalibacterium	-0.93	-2.16	-0.95	ns	-1.23	1.21

Supplementary S10: RFAX results in differences in microbial composition at genus level in **CM 2** after 12 h and 24 h fermentation *in vitro*. Table with the Log2FoldChange for pairwise significantly abundant genera ($p < 0.05$) as output from DESeq analysis. The order of listed genera corresponds to order shown in Figure 6D. ns= non significant

CM 2	Genus	Control vs.		Native vs.
		Native	Extruded	Extruded
12 h	*Acinetobacter*	-11.54	-14.31	-2.76
	Bacillus	-8.28	-7.18	ns
	Clostridium sensu stricto 1	ns	ns	ns
	Faecalibacterium	2.12	3.70	1.58
	Agathobacter	1.33	0.60	-0.73
	Blautia	0.32	ns	ns
	Unkn. Erysipelotrichaceae	6.02	ns	ns
	Coprococcus	2.53	5.05	2.52
	Dorea	0.81	3.34	2.53
	Lachnoclostridium	0.73	1.33	0.59
	Parabacteroides	ns	ns	ns
	Dialister	-0.69	-0.37	0.32
	Unk. Lachnospiraceae	ns	ns	ns
	Enterococcus	-1.75	-2.49	-0.74
	Acidaminococcus	-0.47	0.71	1.17
	Escherichia/Shigella	-1.52	-2.44	-0.93
	Bacteroides	-1.19	-1.72	-0.53
24 h	*Acinetobacter*	-6.75	-9.33	ns
	Bacillus	-6.25	-5.89	ns
	Clostridium sensu stricto 1	-9.20	-9.55	ns
	Faecalibacterium	ns	ns	ns
	Agathobacter	7.67	8.43	ns
	Blautia	ns	ns	1.56
	Unkn. Erysipelotrichaceae	ns	ns	ns
	Coprococcus	ns	4.65	4.05
	Dorea	ns	1.85	1.95
	Lachnoclostridium	-1.05	-1.28	-0.24
	Parabacteroides	-1.29	-1.53	ns
	Dialister	-0.86	-0.58	0.28
	Unk. Lachnospiraceae	ns	ns	ns
	Enterococcus	-0.36	-1.13	-0.78
	Acidaminococcus	ns	0.71	0.63
	Escherichia/Shigella	-0.46	-0.84	-0.38
	Bacteroides	-0.4	-0.7	-0.3
